# Low-dose IL-2 fails to expand Tregs after alemtuzumab: insight into impaired immune tolerance in multiple sclerosis

**DOI:** 10.1093/brain/awag171

**Published:** 2026-05-13

**Authors:** Zoya G Georgieva, Sarah K Howlett, Daniel B Rainbow, Valerie Coppard, Lou Ellis, Lorna B Jarvis, Evangelia Williams, Jennie H M Yang, Bo Sun, Timothy Tree, Alasdair J Coles, Linda S Wicker, Joanne L Jones

**Affiliations:** Department of Clinical Neurosciences, University of Cambridge, Cambridge CB2 0QQ, UK; Department of Clinical Neurosciences, University of Cambridge, Cambridge CB2 0QQ, UK; Department of Clinical Neurosciences, University of Cambridge, Cambridge CB2 0QQ, UK; Department of Clinical Neurosciences, University of Cambridge, Cambridge CB2 0QQ, UK; Department of Clinical Neurosciences, University of Cambridge, Cambridge CB2 0QQ, UK; Department of Clinical Neurosciences, University of Cambridge, Cambridge CB2 0QQ, UK; Department of Immunobiology, King’s College London, London SE1 9RT, UK; Department of Immunobiology, King’s College London, London SE1 9RT, UK; Department of Clinical Neurosciences, University of Cambridge, Cambridge CB2 0QQ, UK; Department of Immunobiology, King’s College London, London SE1 9RT, UK; Department of Clinical Neurosciences, University of Cambridge, Cambridge CB2 0QQ, UK; Centre for Human Genetics, Nuffield Department of Medicine, NIHR Oxford Biomedical Research Centre, University of Oxford, Oxford OX3 7BN, UK; Department of Clinical Neurosciences, University of Cambridge, Cambridge CB2 0QQ, UK

**Keywords:** regulatory T cells, interleukin-2, autoimmunity, multiple sclerosis

## Abstract

Regulatory T cells (Tregs) are essential to preventing autoimmunity. They depend upon interleukin-2 (IL-2) for optimal function and, due to high expression of the CD25 subunit of the IL-2 receptor, are 10-fold more sensitive to IL-2 than effector T cells (Teffs). Consequently, low-dose IL-2 can be used to expand Tregs preferentially, a therapeutic strategy that has shown promise in a number of autoimmune and inflammatory conditions—including graft-versus-host disease and lupus—where IL-2 driven expansion correlates with improvements in some clinical markers of disease activity.

Autoimmunity is a frequent delayed complication of treatment with the lymphocyte-depleting drug alemtuzumab in relapsing-remitting multiple sclerosis (RRMS). Here, using *in vitro* assays, a pre-clinical mouse model and an experimental medicine study, we investigated whether low-dose IL-2 could selectively expand Tregs in alemtuzumab-treated RRMS patients.

Six months after alemtuzumab treatment, the frequency of patient-derived naïve CD4^+^ Teffs expressing high-affinity IL-2 receptors increased from 30.11% ± 5.09% to 72.88% ± 5.57%, and the density of receptors per cell increased, rendering them six times more sensitive to IL-2 *in vitro*, at concentrations that typically favour Tregs. Using a human CD52-expressing transgenic mouse model of alemtuzumab treatment, we found that IL-2 could still preferentially expand Tregs, but only when administered at a later time point, corresponding to more than 6 months post-treatment in patients.

Guided by these findings, we evaluated low-dose IL-2 for Treg expansion in a prospective open-label mechanistic study of RRMS patients who had received alemtuzumab more than 6 months previously. IL-2, at a dose and frequency similar to that previously shown to expand Tregs in autoimmune diabetes (0.3 × 10^6^ IU/m^2^ twice a week), was well tolerated and safe, but unexpectedly failed to expand Tregs.

We discuss the potential reasons underlying this lack of response, ruling out sIL2RA-related neutralization and instead considering ceiling effects on Treg proliferation, Treg exhaustion and intrinsic Treg dysfunction in MS as possible contributors. Together, our findings demonstrate that low-dose IL-2 alone is not an effective strategy for promoting Treg expansion post-alemtuzumab and is therefore not a viable approach on its own for preventing post-treatment autoimmune complications.

## Teaser

Georgieva *et al.* investigated whether interleukin-2 could expand regulatory T cells (‘peacekeeping’ immune cells) to reduce autoimmune complications after alemtuzumab treatment in MS. Despite success in other conditions, this approach failed in MS, providing insights into mechanisms underlying immune tolerance breakdown.

**See Hunt (https://doi.org/10.1093/brain/awag308) for a scientific commentary on this article.**

## Introduction

Multiple sclerosis (MS) is an autoimmune disease of the CNS, characterized by the immune-mediated destruction of myelin sheaths, leading to the accumulation of neurological deficits and progressive disability. Early treatment with highly effective disease-modifying therapy has been associated with delayed progression of disability and better outcomes.^1^

Alemtuzumab is a lymphocyte-depleting humanized monoclonal antibody against CD52, a protein expressed on mature lymphocytes, monocytes and dendritic cells. Administered in two courses given 12 months apart, it is classified as an ‘induction therapy’ due to its ability to provide long-term efficacy without continuous treatment.^2^ Although highly effective, alemtuzumab is usually reserved as a second- or third-line therapy, largely due to its potential to induce secondary autoimmune diseases, including thyroid disorders (affecting 40% of treated patients), idiopathic thrombocytopaenic purpura (1%–2%)^3^ and, more rarely, other autoimmune conditions.

The mechanisms of secondary autoimmunity are not fully understood. Following treatment, B cells recover rapidly through the generation of new cells in bone marrow, while T-cell recovery is slow and occurs, at least initially, through the proliferation of residual cells (a process termed lymphopaenia-induced proliferation), rather than via thymopoiesis.^4^ Repopulation kinetics^4,5^ or major phenotypic differences^6^ have not been observed in those who develop autoimmunity, but affected individuals have a more restricted post-treatment T-cell repertoire^4^ with expansion of pre-existing clones,^6^ implying a lack or loss of regulatory control.

In mouse models, lymphopaenia-induced proliferation promotes autoimmunity when combined with additional factors, such as the loss of regulatory T cells (Tregs)^7^—a subset of CD4 T cells critical for suppressing self-reactive immune responses and known to be functionally impaired in people with MS^8^—and tolerance can be restored by their adoptive transfer.^9^

As well as depleting conventional T cells, alemtuzumab also depletes Tregs, although their frequency within the CD4+ T-cell pool transiently increases for 3–6 months after each treatment cycle, before returning to baseline.^4^ By 2–3 years—the peak period of autoimmune risk—Treg frequency is normal, but absolute numbers remain low.^5^ Combined with their known, potentially persistent functional impairment,^10^ we reasoned that persistently low Treg numbers might contribute to secondary autoimmunity and hypothesize that augmenting Tregs during post-alemtuzumab immune reconstitution might mitigate the risk.

Human Tregs are characterized by stable FOXP3 expression due to demethylation of the Treg-specific demethylated region (TSDR) within *FOXP3*,^11^ low expression of the IL-7 receptor (CD127) and expression of the high-affinity IL-2 receptor—consisting of the CD25, CD122 and CD132 subunits. CD25 captures free IL-2, facilitating receptor engagement,^12^ while CD122 and CD132 expression are required for signal transduction; CD122 is downregulated following receptor engagement.^13^ Intermediate-affinity receptors (lacking CD25) are widely expressed, but the high-affinity receptor is constitutively expressed at high levels on Tregs, CD56^bright^ natural killer (NK) cells and memory CD4 effector T cells (Teffs); a small subset of naïve CD4 Teffs also express CD25 but to much lower levels.^14^ Overall, Tregs respond to IL-2 at 10-fold lower concentrations compared to other immune cells,^15^ and they require it for optimal function.^16,17^

Since low-dose IL-2 (ldIL2) can preferentially promote human Treg expansion, potentially accompanied by enhanced Treg suppressive capacity^18^ and with limited effects on other lymphocyte populations, it is a promising therapeutic strategy for restoring immune tolerance. Doses of 0.5–1.5 × 10^6^ IU have been used to preferentially expand Tregs in different disease contexts, including graft-versus-host disease,^19^ lupus,^20^ inflammatory bowel disease,^21^ MS^22^ and autoimmune diabetes,^23-25^ irrespective of their diverse underlying pathophysiologies.^26^ IL-2 has been used in the context of immune reconstitution, e.g. in graft-versus-host disease^19^ at low doses aimed at expanding Tregs, and at higher doses post-chemotherapy^27^ and in HIV, aiming to support bulk CD4 T-cell reconstitution but also causing the expansion of CD4^+^CD25^+^ Treg-like cells.^28^ However, low-dose IL-2 has not been tested in the unique context of immune reconstitution following alemtuzumab treatment for MS.

Our study aimed to establish whether ldIL2 can expand Tregs in MS patients following alemtuzumab treatment as a potential strategy for mitigating secondary autoimmunity. Using *in vitro* techniques and a mouse model, we first characterized the Treg phenotype and IL-2 signalling pre- and post-alemtuzumab, before testing Treg expansion with ldIL2 in a small open-label experimental medicine study involving MS patients who had received alemtuzumab as part of their routine clinical care.

## Materials and methods

### Donors and ethics statement

Informed consent was obtained from all participants providing samples for *in vitro* assays under ethics REC-11/33/0007. The patients and healthy control donors contributing samples to the *in vitro* assays in this study are described in Supplementary Table 1.

### Cell isolation and storage

Human peripheral blood mononuclear cells (PBMCs) were isolated by Ficoll density gradient centrifugation, frozen in cell freezing medium at −80°C overnight, then stored in liquid nitrogen. For use, vials were thawed in a 37°C water bath for 1 min, diluted 1:10 with pre-warmed complete medium [X-VIVO-15 + 10% fetal calf serum (FCS)], centrifuged, and re-suspended as required.

### Human flow cytometry

Human cells were blocked with 2% mouse serum and Fc receptor block for 15 min. If applicable, chemokine receptor staining was performed at 21°C for 15 min, followed by surface staining using specified antibodies (Supplementary Table 2) and TrueStain monocyte blocker in staining buffer (2.5% FCS, 1% penicillin-streptomycin) for 45 min at 4°C. Zombie Fixable viability dyes or Live Dead Blue were used for viability discrimination, staining for 20 min at 4°C (for cell sorting, 1:20 7-AAD was used). Intracellular staining employed the FOXP3 permeabilization kit (Invitrogen) according to the manufacturer’s instructions, followed by intracellular staining for 60 min at 4°C. Quantitative flow cytometry included the acquisition of 8-peak MEFL beads (Spherotech, lot AL01) acquired with BD Application settings, alongside the stained samples. Bead standards and BD Application settings controlled for daily instrument variation, ensuring fluorescence intensity reflects true biological changes.

Where indicated, cell sorting was performed using a BD FACSAria or Influx sorter. Spectral cytometry was performed on a Cytek Aurora five-laser cytometer using Cytek Application Settings (QC bead lot: 2003).

### Mouse flow cytometry

Mouse blood (15–30 μl) was diluted in staining buffer, stained with primary fluorophore- or biotin-conjugated antibodies for 45 min (then streptavidin if applicable), washed, stained with Zombie Fixable dye for 20 min at 4°C and, if applicable, subjected to intracellular staining as described earlier. Surface-stained cells were instead fixed in 0.25% paraformaldehyde for 15 min at 4°C. Samples were acquired on a BD Fortessa with 8-peak bead standards within 24 h.

### pSTAT5 signalling

Samples were stained in multiplex to eliminate technical variability, as previously described.^29^ Briefly, PBMCs were rested in X-VIVO-15/1% human AB serum for 30 min at 21°C, stained with Zombie Viability dye, washed, and resuspended at 3 × 10^5^ cells/100 µl. PBMCs were IL-2 stimulated (Proleukin, 30 min, 37°C), fixed on ice, then washed. Samples were incubated with PhosFlow Perm Buffer III, then barcoded, and combined in a single tube for staining (50 min on ice; antibodies in Supplementary Table 2). Samples were washed and acquired on a BD Fortessa with Application Settings within 24 h.

### IL-2 and sIL2RA measurement

IL-2 and sIL2RA were measured in patient-derived sera pre- and post-treatment with alemtuzumab using the MSD S-PLEX assay (lower limit of quantification: 21 fg/ml) and MSD U-PLEX assay (lower limit of quantification: 10 pg/ml), respectively, at the Department of Clinical Biochemistry (Addenbrooke’s Hospital) in duplicate. Samples were excluded if replicates differed by >25%.

### Measurement of TSDR demethylation

The methylation status of the *FOXP3* TSDR was measured as previously described.^30^ Briefly, sorted Tregs were processed with the Qiagen Epitect Fast Bisulfite kit, which lyses the cells and performs bisulfite conversion in a single step. First round PCR targeted nine CpG sites within the *FOXP3* TSDR, and a second round PCR added index sequences and Illumina-compatible ends, allowing sequencing on an Illumina MiSeq (2 × 300 bp reads). The per cent *FOXP3* demethylation was calculated as the proportion of sequencing reads that contained eight or nine of the CpG sites within the TSDR that were demethylated, relative to the total number of sequencing reads. Since there is a known bias in PCR efficiency of demethylated DNA,^31^ the raw values were corrected according to a standard curve prepared by sorting known proportions of Tregs and Teffs. Its equation was $y=[x-((-0.0012x^{2})+0.1185x)]$. For female donors, the PCR bias-corrected TSDR demethylation values were further multiplied by two to account for the fact that one X chromosome is fully methylated due to X inactivation. This is required because *FOXP3* is located on the X chromosome. Despite PCR bias correction, this can lead to values of over 100% in some female donors.

### Nanostring

RNA was extracted from 100 000 sorted memory (CD45RA^−^) Tregs from four donors pre- and four donors 6 months post-alemtuzumab, using the Zymo Quick RNA microPrep kit. Gene expression was measured with the Nanostring CAR-T panel, detecting up to 780 genes related to phenotype, activation, metabolism, exhaustion and T-cell receptor (TCR) expression. Probe detection was performed on an nCounter station. Raw RNA counts were normalized in nSolver v4.0 using 9/10 housekeeping genes (*OAZ1* excluded). Normalized counts were thresholded at 3 × standard deviation (SD) of the negative control genes (34 counts) in Excel. Genes expressed above threshold in ≥3/4 samples per group were included, excluding TCR genes. Low-abundance transcripts (<50 copies) with disproportionately high fold-changes were excluded. Heat maps were generated in R v4.5 (gplots). A false discovery rate (FDR) of 0.2 was selected for log_2_-fold changes, preserving known Treg-Teff differences. GSEA v.4.2.1 with the Hallmarks set assessed pathway enrichment using all detectable genes after the above filtering process, post- versus pre-treatment.

## CD52 transgenic mouse

A colony of homozygous human CD52-expressing mice on a C57BL/6 background was established by transferring cryopreserved embryos, provided by Sanofi, into pseudo-pregnant females. In this system, human CD52 is expressed on mouse immune cells, enabling lymphodepletion with alemtuzumab, which does not recognize mouse CD52. Homozygosity was confirmed by qPCR using the mouse *CTLA4* gene as a reference, and human CD52 protein expression was verified by flow cytometry on spleens from transgenic mice. Mice (8–12 weeks of age) received 1 mg/kg alemtuzumab intraperitoneally (i.p.) to deplete lymphocytes. Lymphocyte repopulation was assessed by comparing alemtuzumab- and vehicle-treated groups at multiple time points and by serial blood sampling over 20 weeks in four alemtuzumab-treated mice. Mice then received IL-2 [100 000 IU/day subcutaneously (s.c.) for 4 days] at either 2 or 8 weeks post-alemtuzumab treatment. Blood immunophenotyping was performed before and 24 h after IL-2. Results represent combined data from at least two independent experiments.

### Mechanistic human study of low-dose IL2 (LITMUS)

#### Ethics statement and study population

Individuals with RRMS, who had received alemtuzumab as standard treatment from the NHS at least 6 months previously, were recruited to the study. Informed consent was obtained from all participants under ethics 21/WA/0378 (Wales REC 7). Key exclusion criteria were: significant systemic comorbidities; active infection; uncontrolled autoimmune thyroid disease; concurrent/recent use of other immunomodulatory or cytotoxic medication. Pre-existing non-MS autoimmunity was not an exclusion criterion, provided that disease was clinically stable.

#### Intervention

Study participants were treated with six consecutive s.c. injections of Proleukin (0.3 × 10^6^ IU/m^2^ twice per week for 3 weeks). Peripheral EDTA blood was collected at screening and again within 2 weeks at the baseline visit (two pre-treatment samples); then again 4 days after completing Proleukin treatment; and 4 to 5 weeks after completing Proleukin treatment. Absolute Treg cell count and frequency were measured using clinical-grade flow cytometry at the Department of Immunobiology, King’s College London.

#### Primary outcome

The primary end point was the percentage change in Treg frequency (i.e. proportion of CD4 T-cells) from the baseline visit to the value measured 4 days after the final dose administration within the same participant. This was calculated as:


(1) $$\text{\%}Change=100\times \frac{Treg\;freq(Day\;21)-Treg\;freq(Day\;0)}{Treg\;freq(Day\;0)}$$

A within-participant before-after design was adopted to minimize the effect of inter-individual variation in Treg frequency and immune reconstitution.

#### Exploratory outcomes

Exploratory end points included: absolute Treg count; percentage change in absolute Treg count; CD25^hi^FOXP3^hi^ Treg frequency of total CD4 T cells and percentage change thereof (according to Eq. 1), absolute counts of CD4 Teffs, CD8 Teffs, NK cells and B cells; CD25 MFI on Tregs and CD4 Teffs. Safety, tolerability and adverse events were recorded.

#### Clinical-grade flow cytometry for Treg enumeration

Clinical-grade cytometry to measure absolute Treg counts and frequencies was performed on 100 µl of fresh EDTA blood, stained with two panels of antibodies: (i) a modified Beckton Dickinson TBNK reagent Trucount™ tube to identify and determine the percentages and absolute counts of T, B, NK and CD4^+^CD25^+^CD127^lo^ Tregs (TBNK/Treg count); and (ii) a modified Beckman Coulter DURAClone Treg tube to assess FOXP3^+^ Tregs and NK cell subsets. Details of the panels and indicative gating strategies are shown in Supplementary Table 2 and Supplementary Fig. 1. Tubes were processed according to the manufacturer’s instructions, acquired on a Navios flow cytometer and analysed using Kaluza software (Beckman Coulter). Gating analysis was performed by independent analysts.

#### Preparation and handling of IL-2

Proleukin was prepared under aseptic conditions at Cambridge University Hospitals NHS Foundation Trust clinical studies pharmacy and supplied in a 1 ml capped syringe with a shelf life of 7 days at 2°C–8°C, transported under temperature-controlled conditions.

#### Experimental study power calculations

At the time of our study design, there were no published pharmacodynamic studies of ldIL-2 in MS. We therefore based our power calculations on the on raw data from pharmacodynamic studies in autoimmune diabetes,^25^ which showed that six consecutive injections of IL-2 given every 3 days at 0.32 × 10^6^ IU/m^2^ are sufficient to observe a change in Treg frequency and to detect non-Treg cell activation, if present (Supplementary Fig. 2). Assuming an equivalent response in the MS population, five individuals would provide more than 80% power to detect a 46% (±20% SD) change in Treg frequency versus baseline, while accounting for the changes to Treg frequency in the absence of IL-2 (we assumed up to 11% ± 5.7% SD, based on patients with autoimmune diabetes). For patients with MS post-alemtuzumab, the estimated change in Tregs in the absence of any intervention, over the period of Months 9–12 post-alemtuzumab was −6.8% ± 9.7% SD, calculated using raw data from the ‘alemtuzumab-only’ group in the clinical trial of alemtuzumab +/− palifermin^32^), using a paired samples two-tailed *t*-test. The equivalent effect size was 1.96. To account for a potentially smaller than predicted effect size, we initially planned to recruit seven participants.

### Data analysis and statistics

Data were analysed in GraphPad Prism v10. Statistical tests are indicated in each figure legend. In mouse experiments, repeated measures data were analysed using a mixed-effects model with restricted maximum likelihood (REML), to account for small unbalanced group sizes, potential variance heterogeneity and the presence of a shared pre-treatment across groups, which may introduce dependency. Sidak’s correction was applied for multiple *post hoc* comparisons, and significance was defined as *P* < 0.05. In the human experimental medicine study, the primary outcome was assessed by a paired Wilcoxon test; other outcomes were tested by repeated measures ANOVA. All tests were two-tailed.

All results are reported as mean ± standard error of the mean (SEM) unless specified. Cytometry fluorescence intensities are reported as geometric mean (gMFI) ± SEM. unless specified.

## Results

### Human Tregs are activated 6 months post-alemtuzumab in response to endogenous cytokines including IL-2

We have previously reported an increase in the frequency of CD4^+^CD25^hi^ T cells in the first 9 months after alemtuzumab treatment,^4^ but absolute Treg numbers are extremely low.^5^ As a starting point, we characterized the Treg compartment to gain insight into the cells that might be expanded with low-dose IL-2.

Identifying human Tregs is challenging due to the absence of unique markers. Activated Teffs transiently express low levels of FOXP3 and moderate levels of CD25,^33^ without acquiring suppressive properties, and show reduced CD127 expression during lymphopaenia-induced proliferation.^34^ A more reliable Treg identifier is demethylation of the *FOXP3* TSDR, which indicates stable expression of *FOXP3* and is absent in activated Teffs.^11^

First, to confirm that memory FOXP3^hi^ T cells, which predominate post-alemtuzumab, are *bona fide* Tregs, CD45RA^−^FOXP3^hi^ and FOXP3^−^ CD4 T cells (gated as shown by Miyara *et al*.^35^) were fluorescence-sorted from donors pre-treatment and at Months 1–3, 6 and 12 after the first cycle of alemtuzumab, alongside age-matched healthy controls (*n* = 7; gating strategy in Supplementary Fig. 3). Memory CD45RA^−^FOXP3^hi^ cells from patients exhibited a fully demethylated TSDR, comparable to healthy controls, at all time points post-alemtuzumab, confirming that they are *bona fide* Tregs (Fig. 1A). As expected, the FOXP3^−^ cells were methylated at this region.

**Figure 1:**
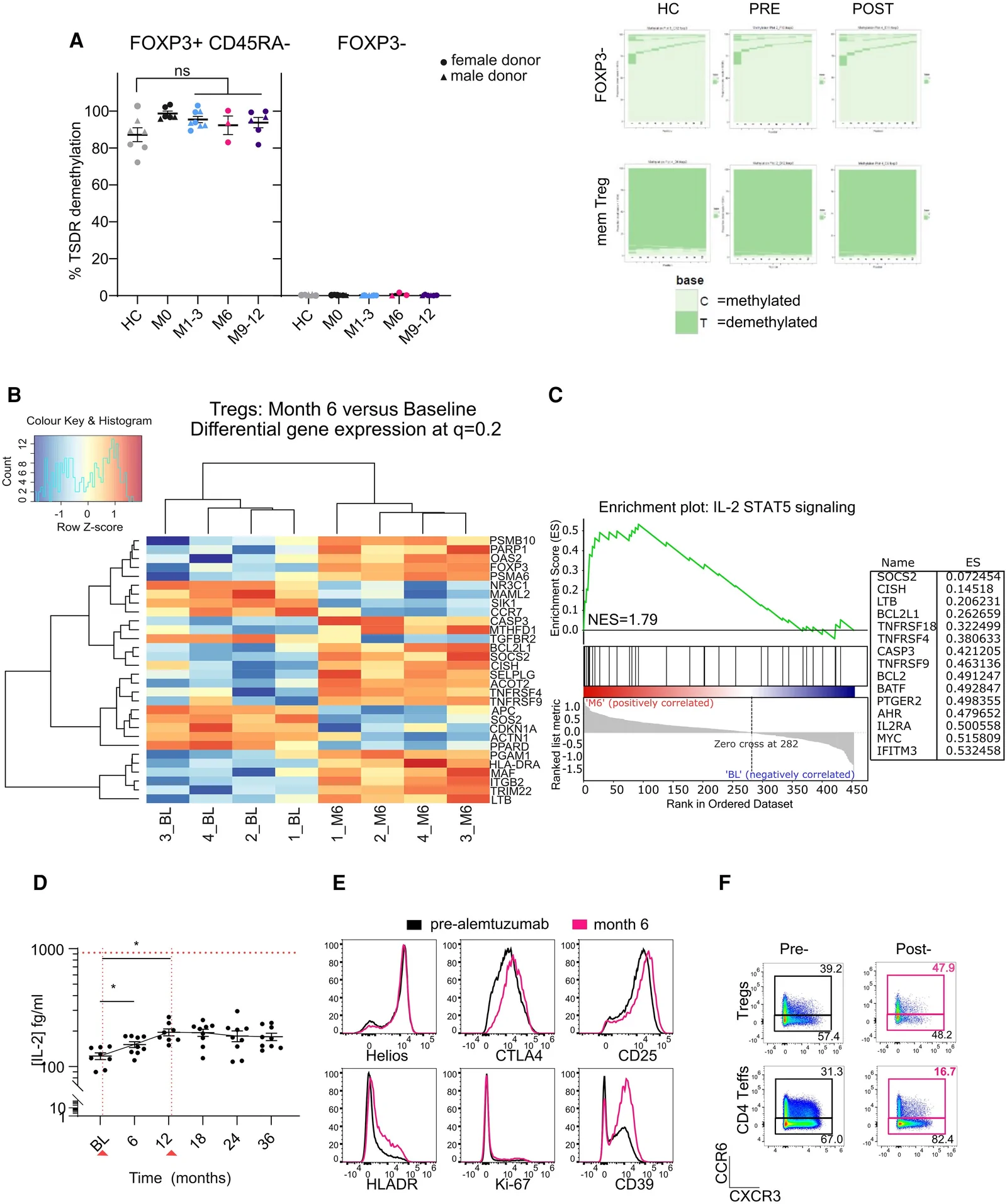
**Human memory FOXP3^hi^ T cells post-alemtuzumab are *bona fide* regulatory cells and appear highly activated.** (**A**) Demethylation of the *FOXP3* gene indicates its stable expression. Methylation status was assessed in FOXP3^hi^CD45RA^−^ T cells from different individuals with multiple sclerosis sampled pre- and at the indicated times post-alemtuzumab, and from seven healthy controls (HC). TSDR demethylation was not significantly different between HC and any post-alemtuzumab time point (one-way ANOVA with Sidak’s multiple comparisons). The equivalent region in FOXP3^−^ cells was fully methylated. (**B**) Top 30 differentially expressed genes in CD45RA^−^ CD127^lo^CD25^hi^ CD4 T cells (memory Tregs) 6 months post-alemtuzumab (*n* = 4 pre- and 4 post-treatment cross-sectional donors) are involved in cell cycle, activation and response to cytokines. Blue denotes transcript downregulation at 6 months. False discovery rate, q = 0.2. (**C**) Enrichment of genes in the IL-2 pathway 6 months post-alemtuzumab shown by gene set enrichment analysis. The green curve represents the cumulative enrichment score. A positive score denotes higher expression post-alemtuzumab. The indicated genes from the IL-2 pathway are ranked near the top of the ranked gene list, arranged by differential expression. NES = normalized enrichment score accounting for the gene set size. (**D**) Serum IL-2 levels increased post-treatment but remained below the level previously shown to activate Tregs *in vitro*, indicated by the horizontal dotted line.^24^ Red arrows and vertical dotted lines indicate alemtuzumab treatment. *n* = 9; restricted maximum likelihood; *F*(3.487,26.50) = 5.260; Dunnett’s test; two missing values due to technical error and intercurrent infection. (**E**) Representative normalized flow cytometry plots from the same nine donors show increased canonical Treg markers and (**F**) preserved CCR6 expression on Tregs post-alemtuzumab. Additional phenotyping data and individual donor-level breakdown are in Supplementary Fig. 5. *P < 0.05. Treg = regulatory T cell; TSDR = Treg-specific demethylated region.

Next, memory Tregs (CD45RA^−^ CD127^lo^CD25^hi^ CD4^+^ T cells) were fluorescence-sorted from four donors with untreated RRMS and four donors 6 months post-alemtuzumab treatment (Supplementary Fig. 4 for sort gating strategy). Gene expression was measured using the Nanostring CAR-T panel, which quantifies 780 genes encompassing T-cell activation, TCR chain, migration, metabolism and exhaustion. Of 670 non-TCR genes, 454 were detectable above threshold, of which 30 were differentially expressed at Month 6 versus pre-treatment (FDR = 0.2). These genes were linked to cell cycle progression (*CDKN1A*), survival and apoptosis (*BCL2L1*, *CASP3*, *PARP1*), activation (*HLA-DRA*), cytoskeletal structure and cell trafficking (*ACTN1*, *SELPLG*, *PGAM1*, *ITGB2*) and lipid metabolism (*PPARD*, *ACOT2*) (Fig. 1B). Notably, *ITGB2*, encoding a component of LFA-1 (CD11a/CD18) which permits CNS entry^36^ independent of CD49d (the target of natalizumab^37^) was upregulated post-alemtuzumab.

To identify active pathways, gene set enrichment analysis (GSEA) was performed on all 454 Treg-detectable genes using the Hallmarks gene set. Memory Tregs post-alemtuzumab showed enrichment of genes involved in IFNα (NES 1.74, *P* = 0.011, FDRq = 0.028) and IFNγ pathways (NES 1.54, *P* = 0.017, FDRq = 0.108) with downregulation of TNFα signalling (NES −2.19, *P* < 0.0001, FDRq < 0.0001). This resembles the published gene expression signature of CD4 Teffs undergoing homeostatic proliferation after chemotherapy.^34^ Additionally, genes involved in the response to IL-2, including *CISH*, *SOCS2*, *IL2RA*, and *BCL2L1* (NES 1.79, *P* = 0.005, FDRq = 0.03) (Fig. 1C) were enriched, suggesting endogenous IL-2 signalling.

Using an ultra-sensitive assay, we measured serum IL-2 longitudinally in nine donors, pre- and post-alemtuzumab—at Months 6–9 and 12, and Months 18–21 and 36 post-first treatment. IL-2 levels increased following the first treatment (Fig. 1D), but remained within the reported ‘healthy’ range (350 fg/ml ± 1640 fg/ml SD^38^).

Our gene expression analysis and previous work^4^ suggested that Tregs post-alemtuzumab are activated. To confirm this, high-parameter spectral flow cytometry was performed on samples from the same nine individuals. At Month 6, HELIOS expression—indicative of stable Treg identity^39^—was maintained, consistent with our TSDR demethylation findings. Classical Treg markers CD25, CTLA-4 and CD39 were increased 6 months post-alemtuzumab treatment (Fig. 1E), as were HLA-DR and Ki-67. CCR6, implicated in CNS entry,^40^ was retained on Tregs but not on CD4 Teffs (Fig. 1F), suggesting a Treg phenotype favourable to controlling MS disease activity. However, we also observed increased expression of markers associated with impaired Treg function in some contexts, e.g. CD49d,^41^ CD226^42^ and PD-1^43^ (Supplementary Fig. 5).

Overall, post-alemtuzumab Tregs exhibited an activated memory phenotype, with increased canonical markers and a transcriptional profile indicative of IL-2 responsiveness. This supported the rationale for Treg expansion with ldIL2. Given the importance of selectively targeting Tregs, we next examined IL-2 responsiveness in the effector lymphocyte compartment.

### Naïve CD4 T-effector cells show enhanced *in vitro* IL-2 responsiveness after alemtuzumab via CD25 upregulation

Following alemtuzumab, lymphopaenia drives widespread T-cell proliferation, leading to a memory-like phenotype, including higher expression of CD25 and CD122.^4^ We predicted this could alter the way lymphocytes respond to IL-2. To better understand which cell types might be affected, we measured CD25 and CD122 expression by quantitative flow cytometry in a cross-sectional cohort of people with MS pre- and at Months 6, 9 and 12 post-alemtuzumab. The gating strategy is shown in Supplementary Fig. 6.

Pre-alemtuzumab, 30.11% (±5.90%) of naïve CD4 Teffs expressed CD25, increasing to 72.8% (±5.57%) at Month 6 and remaining elevated at Month 12. The fraction of naïve CD4 Teffs expressing CD122 also increased. CD25/CD122 double-positive naïve Teffs increased from 3.5% pre-treatment to >15% post-alemtuzumab (Supplementary Fig. 7A), with CD25 (but not CD122) per-cell density increasing (Fig. 2A). Since CD4 naïve Teffs in healthy individuals gain CD25 due to age-associated homeostatic proliferation,^14^ we confirmed no significant age difference between the pre- and post-treatment donors.

**Figure 2:**
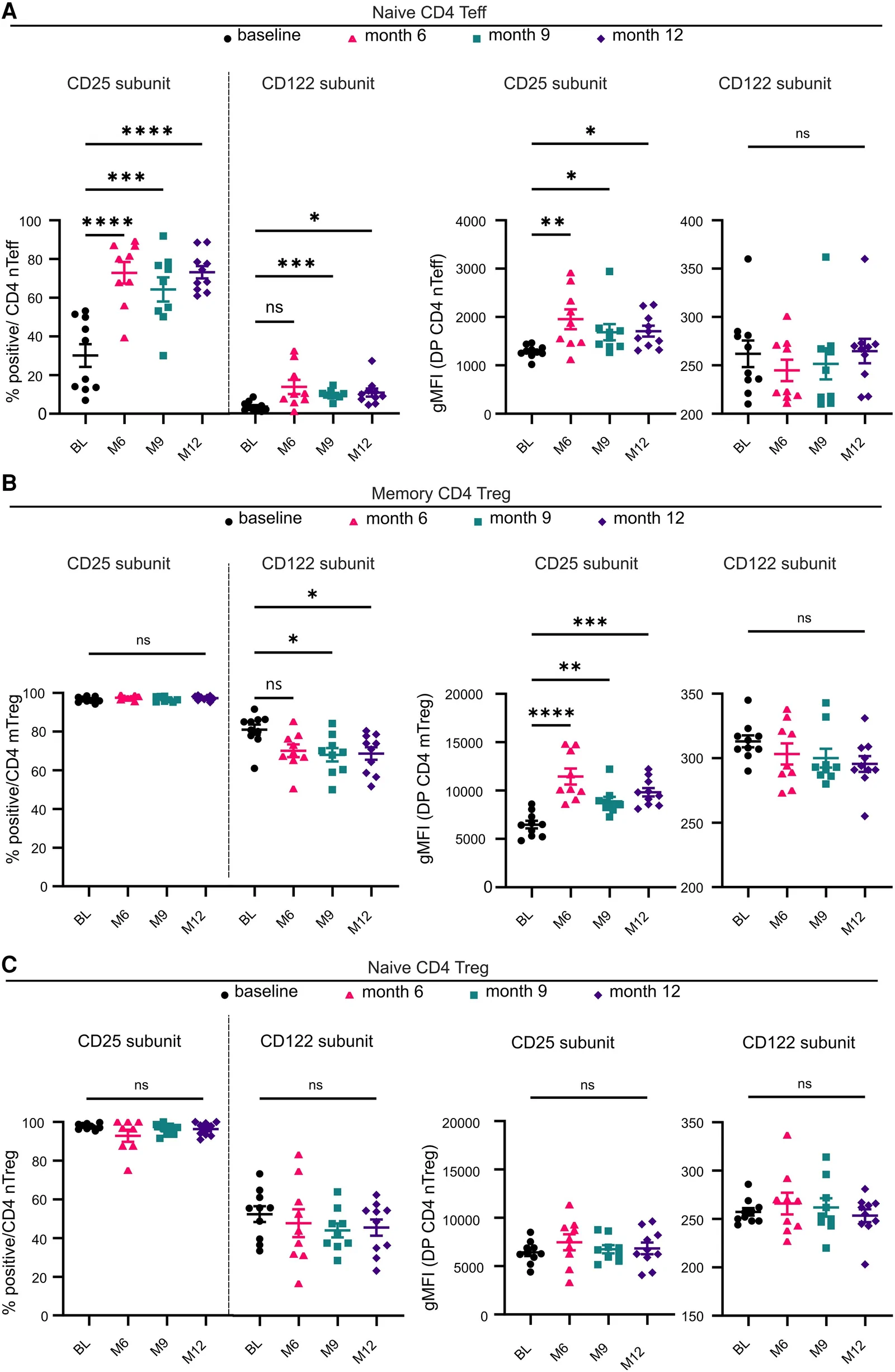
**High-affinity IL-2 receptors significantly increase on naïve CD4 effector T cells after alemtuzumab treatment.** (**A**) Expression of CD25 and CD122 subunits of the IL-2 receptor on human naïve CD4 effector T cells (nTeff), shown as cell frequencies, and density of the receptors per cell (gMFI). (**B** and **C**) Expression of IL-2 receptor subunits on (**B**) memory (mTreg) and (**C**) naïve T regulatory cells (nTreg). Different donors were sampled cross-sectionally at each time point. One-way ANOVA, or where standard deviations are not equal, Welch’s ANOVA with Dunnett’s multiple comparisons test. gMFI data were analysed with Kruskal-Wallis test with Dunn’s multiple comparisons. *P < 0.05, **P < 0.01, ***P < 0.001, ****P < 0.0001. DP = double positive for CD25 and CD122; gMFI = geometric mean fluorescence intensity, which reflects receptor density per cell. Normalization refers to the use of fluorescence bead standards to quantify gMFI.

Post-alemtuzumab, the predominant memory Treg population also increased its expression of CD25 (Fig. 2B). The frequency of CD122^+^ memory Tregs decreased, without detecting a significant change in the per-cell density of this subunit. Given CD122 is required for IL-2 signalling, and is downregulated in response to IL-2, we questioned whether Tregs would remain responsive to IL-2. As expected, few naïve CD4 Tregs were detectable post-alemtuzumab, and the expression of IL-2 receptor subunits did not change on this population (Fig. 2C).

Changes in the memory CD4, CD8, B-cell and NK compartments were less marked (Supplementary Fig. 7B–G). Notably, fewer CD4 memory Teffs expressed CD25 without a significant change in per-cell expression. A minority of CD8 Teffs expressed CD25, mostly in the memory compartment (6.73% ± 1.70% naïve CD8; 26.07% ± 4.77% memory CD8). Although the frequency of CD25^+^CD122^+^ memory CD8 cells declined, CD25 per-cell expression increased, suggesting the potential for enhanced IL-2 responsiveness of this small subset post-alemtuzumab. As B cells reconstitute *de novo* from bone marrow, less mature phenotypes emerge to replace activated B cells,^44^ so as expected, both memory and naïve B cells decreased their expression of CD25 post-alemtuzumab. The frequency of CD56^bright^CD16^lo^ NK cells transiently increased 6 months post-alemtuzumab, but their expression of CD25 and CD122 did not change. Finally, dimensionality reduction analysis, performed using unsupervised (FlowSOM-on-tSNE^45^ on T cells) and semi-supervised methods (FlowAtlas.jl^46^) did not reveal any additional notable alterations in CD25 or CD122 expression (Supplementary Fig. 8).

Thus, the main change observed post-alemtuzumab was an increase in naïve CD4 Teffs expressing high-affinity IL-2 receptors, as well as greater density of these receptors per cell. This raised the possibility that naïve CD4 Teffs may gain the ability to respond to IL-2 at doses previously considered Treg-selective.

To test this, cryopreserved PBMCs from pre- and post-alemtuzumab donors (6 and 12 months post-treatment) were stimulated with IL-2 and phosphorylated STAT5 (pSTAT5) was measured as previously described.^29^ The maximal pSTAT5 response in naïve CD4 Teffs 6 months after treatment rose from 40% pre-treatment to 60% of cells post-treatment, with a six-fold EC50 reduction (1.886 IU/ml to 0.3101 IU/ml; Fig. 3A and Supplementary Fig. 9A–F). However, a corresponding increase in IL-2 responsiveness was not seen in the memory Treg compartment (CD45RA^−^ FOXP3^+^CD25^+^ cells; Fig. 3B). Whereas pre-alemtuzumab 0.25 IU/ml of IL-2 activated <10% of naïve Teffs and virtually all Tregs, post-alemtuzumab this same dose activated >30% naïve Teffs (Fig. 3C). Furthermore, the per-cell pSTAT5 fluorescence intensity in naïve CD4 Teffs also increased modestly, mirroring CD25 (Fig. 3D). Nevertheless, pSTAT5 levels on a per cell basis remained higher in Tregs even after alemtuzumab treatment, and the proportion of responding Tregs greatly exceeded that of CD25+ Teffs (Supplementary Fig. 9G), suggesting Tregs retained higher IL-2 sensitivity. No significant changes were detected in NK cell or naïve CD8 T-cell responsiveness to IL-2 post-alemtuzumab.

**Figure 3:**
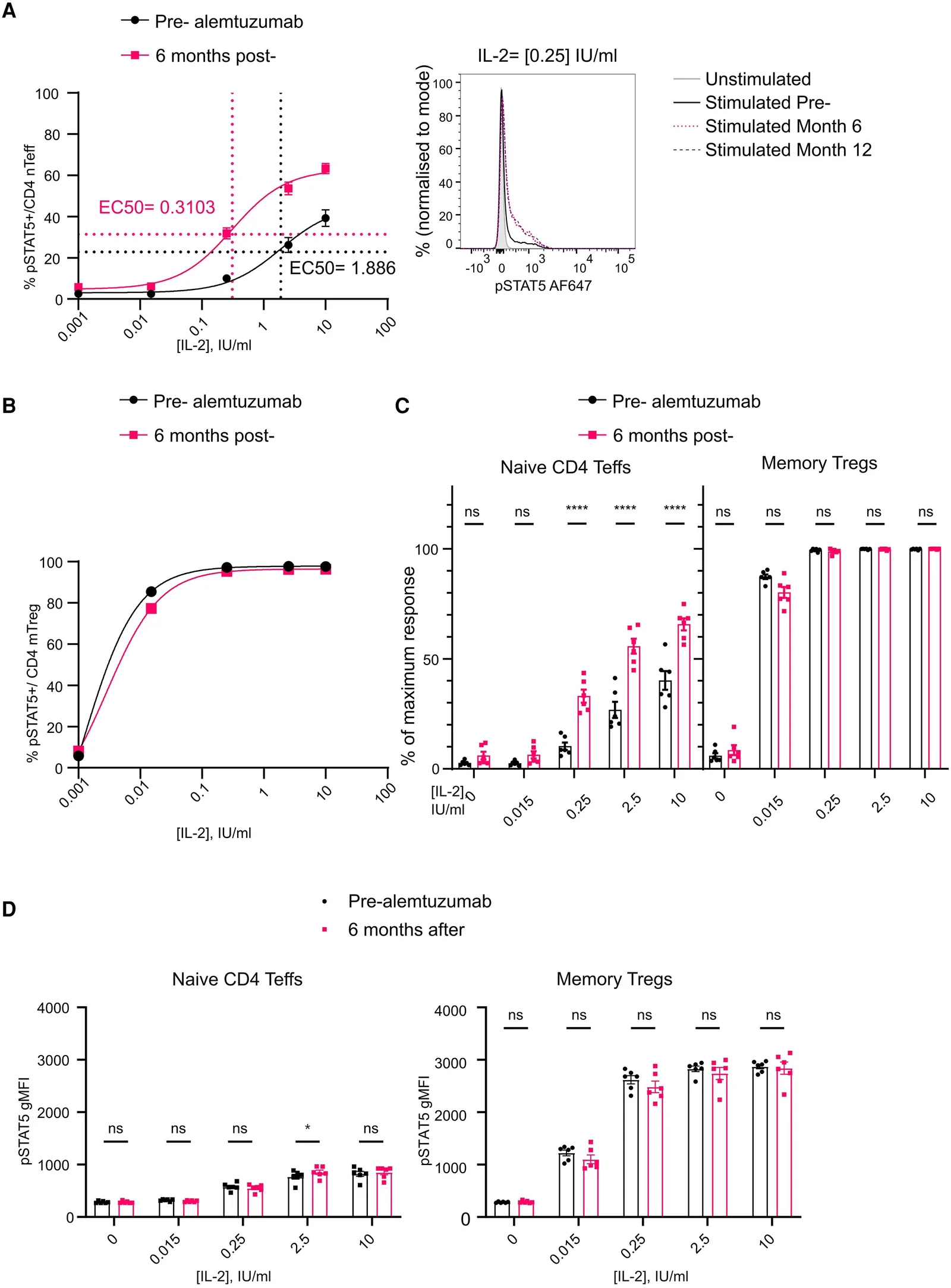
**Human naïve CD4 effector T cells become responsive to lower concentrations of IL-2 after alemtuzumab, while regulatory T cells do not.** (**A**) Response of naïve CD4 effector T cells (Teffs) to increasing concentrations of IL-2 *in vitro*, showing a reduction in EC50. *n* = 6 individuals before and 6 months after the first or second treatment cycle with alemtuzumab. Least squares regression, df = 27, *R*^2^ = 0.85 pre- and *R*^2^ = 0.95 post-alemtuzumab; probability that the curves are different >99.9%. A representative flow cytometry histogram from one donor at different time points is shown on the *right*. (**B**) Memory Tregs did not become more responsive to IL-2, unlike naïve effector cells. *n* = 6 individuals pre- and 6 months post-alemtuzumab, least squares regression, df = 27, *R*^2^ = 0.99 and *R*^2^ = 0.98, respectively. The probability that the curves are significantly different was 99.98%, but the curves diverge below 0.015 IU/ml; concentrations below this level were not tested, so it is not possible to conclude that Tregs are less sensitive post-alemtuzumab. (**C**) The gap between naïve CD4 Teff and Treg responses to IL-2 was narrower post-alemtuzumab. The same data shown in **A** and **B**, normalized to the maximum response of Tregs at each concentration, analysed by two-way repeated measures (RM) ANOVA. Significant main effects of Cell type/IL-2 concentration, Time, and their interaction were detected (all *P* < 0.0001). *Post hoc* Sidak’s test increased responsiveness post-treatment in naïve Teffs at 0.25–10 IU/ml IL-2 (*P* < 0.0001); no significant changes (ns) were observed in memory Tregs. (**D**) Per cell level of the phosphorylated transcription factor pSTAT5 increased after alemtuzumab in both Teffs and Tregs but remained greater in Tregs than Teffs. pSTAT5 gMFI reflects signalling strength per cell and is shown exclusively in the pSTAT5+ subset of cells at each concentration of IL-2. Two-way RM-ANOVA with Time, IL-2 and interaction terms. [IL-2] had a significant main effect [*F*(4,25) = 86.49, *P* < 0.0001]; Time and interaction effects were not significant (ns). Šídák’s *post hoc* test identified a significant increase at 2.5 IU/ml IL-2 (*P* = 0.045). This region is near the EC50 and therefore most sensitive to shifts. No significant differences pre/post-alemtuzumab were identified for Tregs at any IL-2 concentration. *P < 0.05, ****P < 0.0001. EC50 = half-maximal effective concentration; gMFI = geometric mean fluorescence intensity; Treg = regulatory T cell.

These data indicate that the therapeutic window for preferential Treg expansion using ldIL2 may be narrower following alemtuzumab treatment, primarily due to heightened CD4 naïve Teff responsiveness. This poses a safety concern, since expanding non-Tregs in a pro-autoimmune setting is undesirable. However, i*n vitro* models have limitations: (i) transient pSTAT5 activation does not necessarily equate to proliferation; (ii) *in vitro* assays lack the complex immune microenvironment; and (iii) IL-2 pharmacokinetics cannot be fully replicated. So, to assess whether IL-2 could still preferentially expand Tregs *in vivo* without evidence of increased off-target effector activation post-alemtuzumab, we next turned to a mouse model.

### IL-2 preferentially expands Tregs in alemtuzumab-treated human CD52-expressing transgenic mice

Alemtuzumab does not deplete immune cells in wild-type mice due to insufficient CD52 homology, so we established a colony of human CD52-expressing transgenic mice on a C57BL/6 background. Expression of human CD52 was confirmed by flow cytometry (Fig. 4A), and lymphocyte repopulation kinetics were assessed following a single 1 mg/kg dose of alemtuzumab (Fig. 4B and C). Mice were profoundly T-cell lymphopaenic 4 days post-alemtuzumab, persisting at 2 weeks. FOXP3^+^ Tregs were enriched within the CD4 T-cell pool at both timepoints, resembling human blood at 3–6 months post-treatment when lymphopaenia is marked, and Tregs are increased as a proportion of CD4 T-cells (despite both being low in absolute number).^4^ By Week 8 in mice, T-lymphopaenia was no longer significant, and Treg enrichment was absent, resembling humans after 9–12 months.^4^ Hereafter, 2 weeks is termed ‘early’ post-alemtuzumab and 8 weeks as ‘late’ post-alemtuzumab.

**Figure 4:**
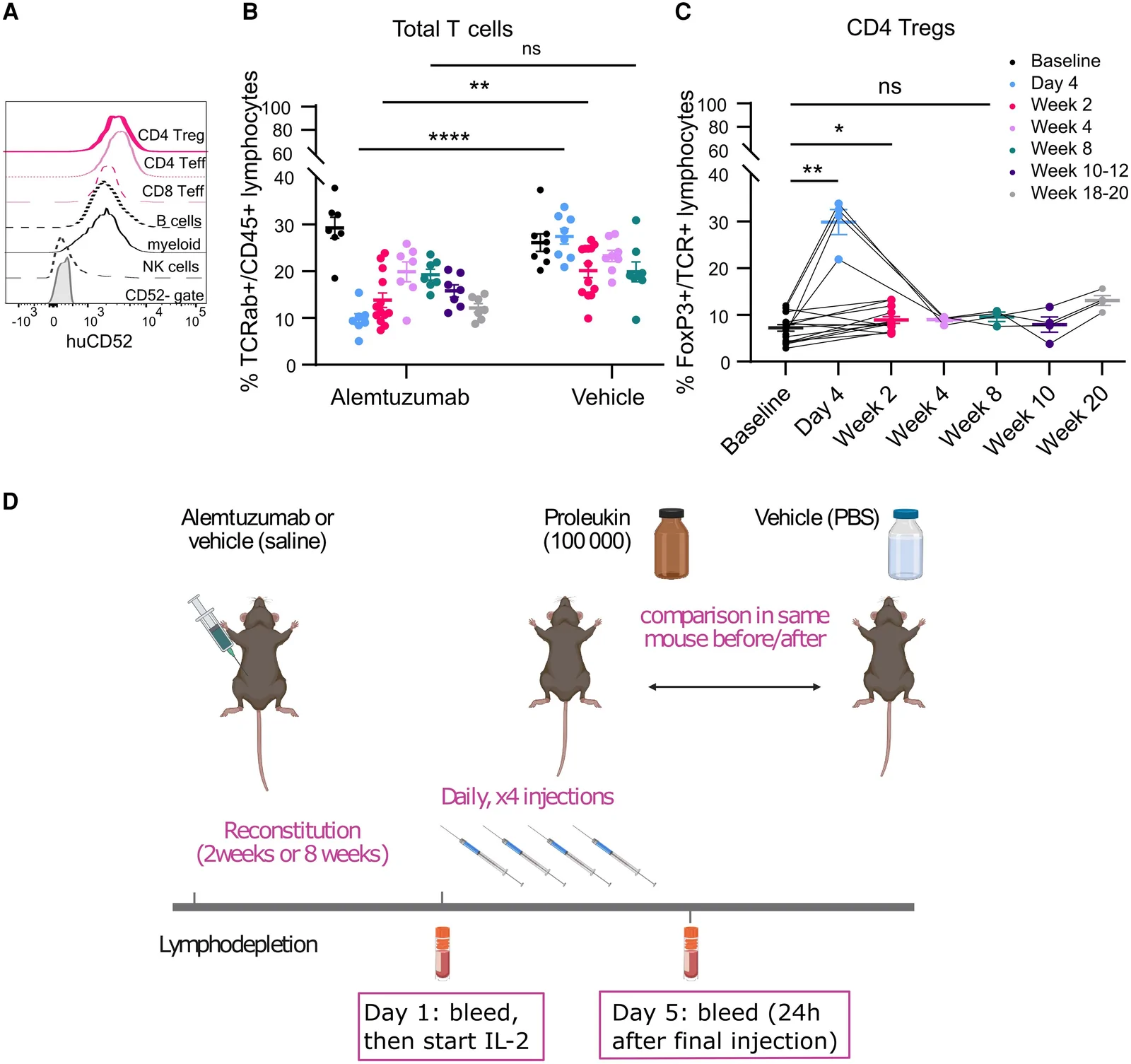
**Human CD52 transgenic mice treated with alemtuzumab recapitulate human immune reconstitution and serve as a model for dosing with IL-2**. (**A**) Human CD52 is expressed on mouse immune cells in a pattern similar to that in humans. (**B**) A single dose of alemtuzumab (1 mg/kg i.p.) at 8–12 weeks of age resulted in T-cell lymphopaenia at Day 4 (*P* < 0.0001) and Week 2 (*P* = 0.0099) compared to vehicle- treated mice. Two-way ANOVA with Treatment and Time as factors; significant main effects of Treatment [*F*(1,76) = 19.76, *P* < 0.0001], Time [*F*(4,76) = 11.51, *P* < 0.0001] and a Treatment × Time interaction [*F*(4,76) = 9.219, *P* < 0.0001]. Šídák’s *post hoc* test. (**C**) Mouse regulatory T cells (Tregs) were proportionally enriched within CD4 T-cells early (up to 2 weeks) after alemtuzumab, similar to those in humans. Data from four longitudinally sampled mice are shown at Day 4, Week 4, Week 8, Week 10 and Week 20, analysed by a mixed effects model due to a missing value [*F*(2.046,5.728) = 32.82, Geisser-Greenhouse correction = 0.409, Dunnett’s multiple comparisons test]. On the same graph, a separate group of mice is shown, sampled only at baseline and again at Week 2 post-alemtuzumab (*n* = 13; paired-samples *t*-test, *t* = 6.078, df = 12, *P* = 0.018). Black lines connect data points from the same mouse. (**D**) Schematic of the experimental format for IL-2 treatment in CD52 transgenic mice. *P < 0.05, **P < 0.01, ****P < 0.0001. Created in BioRender. Georgieva, Z. (2026) https://BioRender.com/s9siqjc.

Dose-finding experiments established that a dose of 100 000 IU Proleukin (IL-2) administered s.c. daily for 4 days was capable of expanding Tregs in mice not treated with alemtuzumab. This frequent and high-dosing regimen was needed due to IL-2’s short half-life in mice.^47^ In all subsequent experiments, blood was sampled pre-IL-2 (Day 1) and on Day 5 (24 h post-final IL-2 dose) (Fig. 4D), and cell frequencies were measured. Treg expansion was defined as an increase in Treg frequency (as a fraction of CD4 T cells)—as it would be in human studies—alongside a concomitant rise in Ki-67^+^ Tregs.

In mice not treated with alemtuzumab, the Treg fraction increased from 6.77% (±0.75%) to 9.65% (±1.08%) following IL-2 dosing, representing a 1.43-fold change. This was accompanied by a significant increase in Ki-67^+^ proliferating Tregs from 30.97% (±3.56%) to 48.27% (±2.50%) (Fig. 5A and B). No significant changes were observed in vehicle-treated mice. The dosing regime also induced some proliferation of effector lymphocytes (Fig. 5C), without any accompanying significant change in the composition of the lymphocyte pool (Supplementary Fig. 10A).

**Figure 5:**
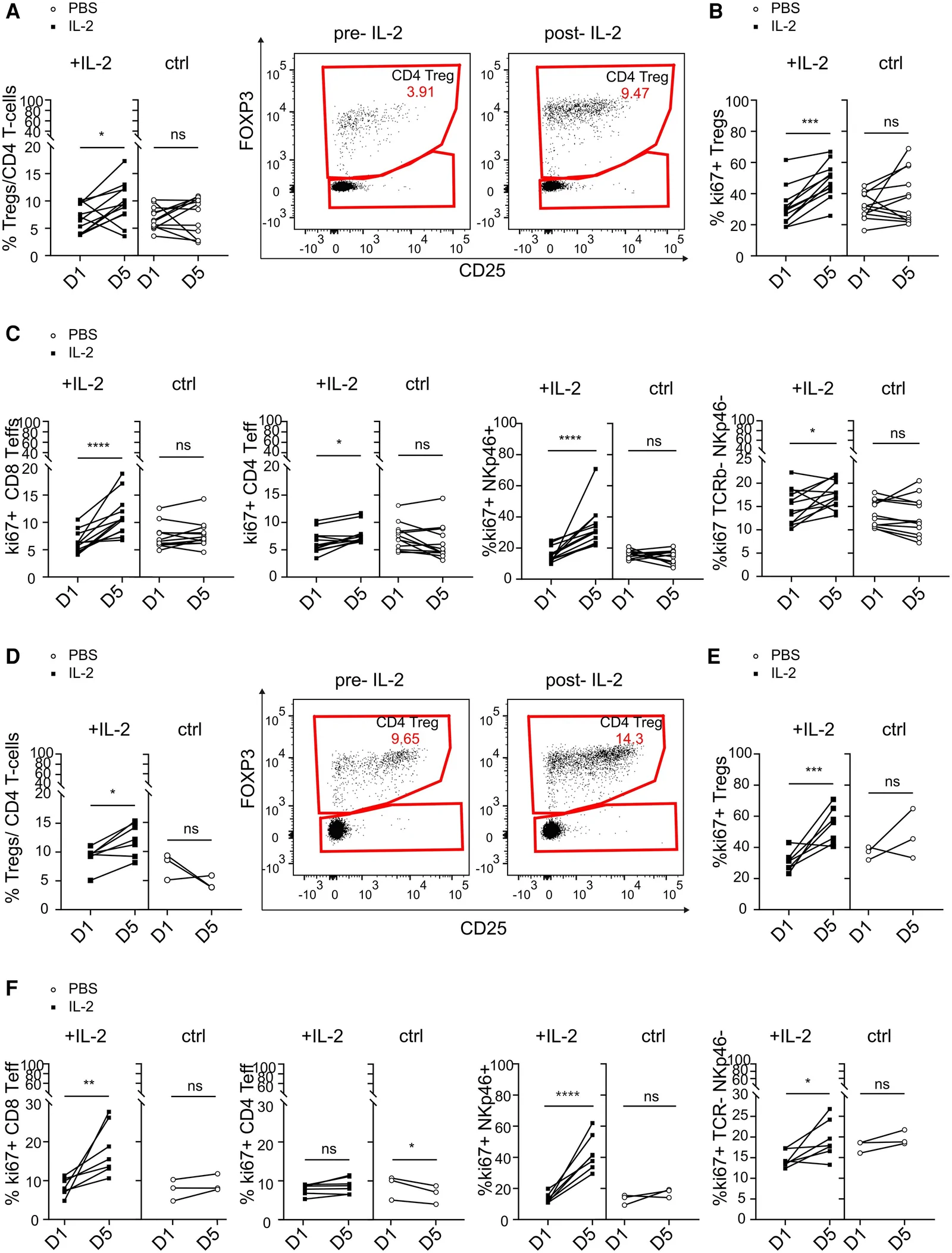
**IL-2 treatment expands regulatory T cells when given late after alemtuzumab.** (**A**) Regulatory T cells (Tregs) increased as a proportion of CD4 T cells after four daily injections of IL-2 in mice not previously treated with alemtuzumab. Representative cytometry dot plots are shown. Restricted maximum likelihood (REML) with fixed factors Time, IL-2 and Time × IL-2, Sidak’s multiple comparisons test. A significant main effect of Time was detected (*P* = 0.035), with an increase in Tregs from Day 1 to Day 5 in the IL-2 group (*P* = 0.025, Sidak’s *post hoc* test). (**B**) The frequency of proliferating Tregs increased in response to IL-2 (same mice as in **A**, analysed by REML; Sidak’s *post hoc* test showed a significant increase in the IL-2 group, *P* = 0.0002). (**C**) Effector lymphocytes also responded to this IL-2 dose in lymphoreplete mice. The TCRβ-NKp46 population predominantly consists of B cells and some NKp46-NK cells. (**D**) In mice treated with alemtuzumab 8 weeks earlier, IL-2 still expanded Tregs as a proportion of CD4 T cells. Representative cytometry dot plots are shown. REML, fixed factors: Time *F*(1,8) = 0.00005, *P* = 0.995; IL-2 *F*(1,8) = 12.16, *P* = 0.0082; Time × IL-2 *F*(1,8) = 13.40, *P* = 0.0064. Sidak’s *post hoc* test: IL-2 group (*P* = 0.0205). (**E**) The frequency of proliferating Tregs increased with IL-2 treatment, 8 weeks after alemtuzumab. REML with fixed factors: Time *F*(1,16) = 12.63, *P* = 0.0026; IL-2 *F*(1,16) = 0.0051, *P* = 0.944; Time × IL-2 *F*(1,16) = 1.47, *P* = 0.243, Sidak’s test: IL-2 group *P* = 0.001. (**F**) IL-2 also causes CD8 effector T cells (Teffs) and natural killer (NK) cells to proliferate in the ‘late’ post-alemtuzumab setting. *P < 0.05, ***P < 0.001; ****P < 0.0001. D = Day.

Next, we tested IL-2 treatment at the ‘late’ post-alemtuzumab time point, corresponding to 9–12 months post-treatment in humans, when Treg ‘enrichment’ wanes, making this a clinically relevant time point. Here, IL-2 increased the Treg fraction from 9.14% (±0.71%) of all CD4 T cells to 12.24% (±1.08%) (Fig. 5D), equivalent to a 1.34-fold increase. Proliferating Treg frequency also increased from 30.91% ± 2.42% to 54.11% ± 4.36% (Fig. 5E). As before, effector populations also proliferated, particularly CD8 Teff and NK cells (Fig. 5F), but again overall lymphocyte proportions were unaltered (Supplementary Fig. 10B).

### IL-2 does not expand mouse Tregs when administered early after alemtuzumab

The optimal timing to treat patients with IL-2 following alemtuzumab is unknown. Therefore, we also examined the effect of IL-2 at the ‘early’ time point—equivalent to 3–6 months post-treatment in humans. Using the same IL-2 dosing regime, we found no increase in Treg frequency as a proportion of CD4 T cells (Fig. 6A), or Treg proliferation (Fig. 6B), indicating a failure of Treg expansion.

**Figure 6:**
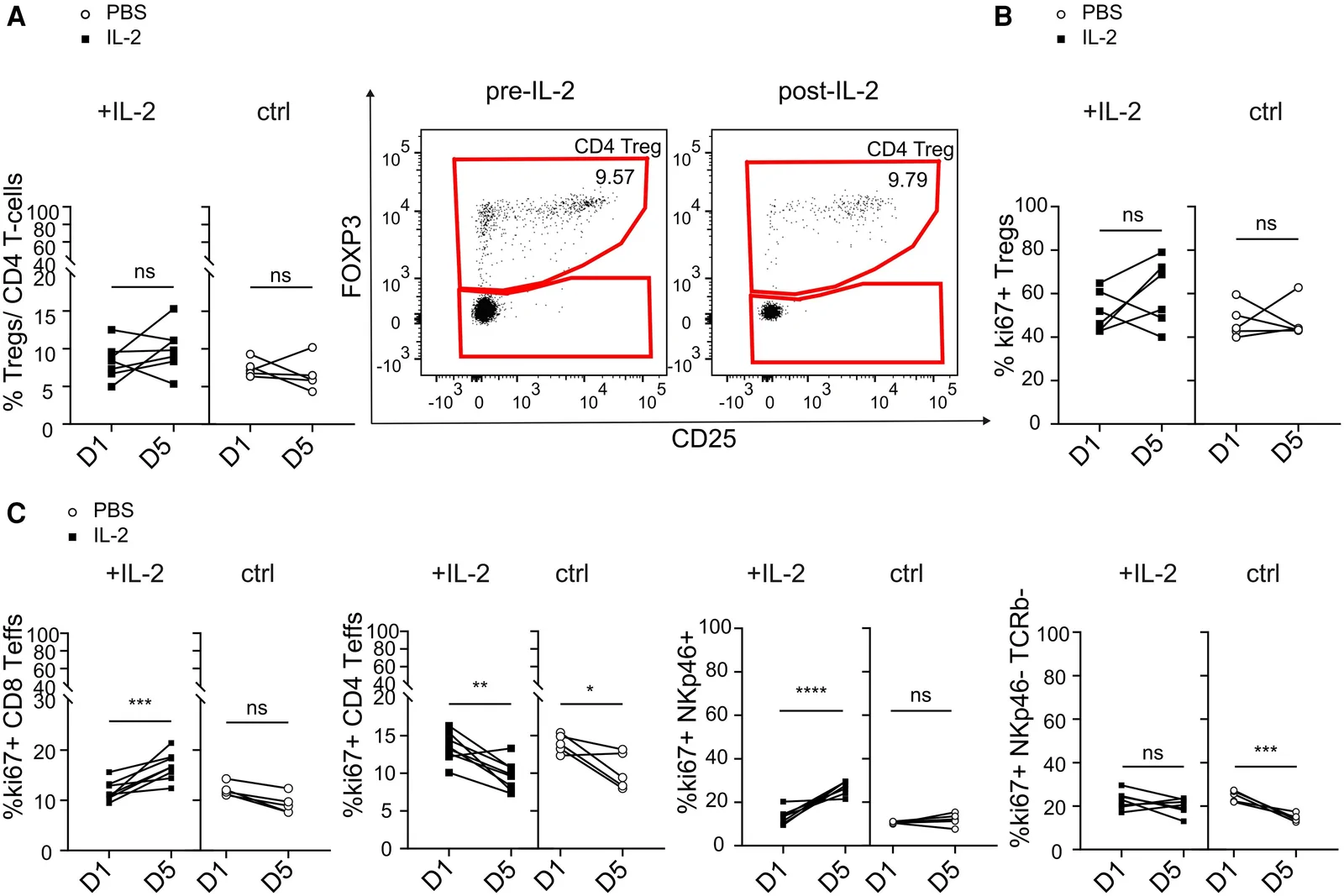
**IL-2 given early after alemtuzumab does not expand regulatory T cells but still activates Teff cells in human CD52 transgenic mice.** (**A**) Regulatory T cells (Tregs) did not expand in response to IL-2 given 2 weeks after alemtuzumab. Representative cytometry dot plots are shown. Restricted maximum likelihood (REML) with fixed factors: Time *F*(1,10) = 0.16, *P* = 0.695; IL-2 *F*(1,10) = 4.40, *P* = 0.062; Time × IL-2 *F*(1,10) = 1.81, *P* = 0.208. Sidak’s test: no significant change (ns) from Day 1 to Day 5 in IL-2 (*P* = 0.369) or control group (*P* = 0.799). (**B**) Treg proliferation did not increase with IL-2. REML: Time *F*(1,8) = 2.07, *P* = 0.188; IL-2 *F*(1,8) = 2.89, *P* = 0.127; Time × IL-2 *F*(1,8) = 1.94, *P* = 0.202. Sidak’s *post hoc* test: no significant change (ns) from Day 1 to Day 5 in either IL-2 or control group. (**C**) Proliferating CD8 effector T cells (Teffs) and natural killer (NK) cells increased in response to IL-2, while CD4 Teff proliferation slowed down irrespective of IL-2 treatment. *P < 0.05, **P < 0.01, ***P < 0.001, ****P < 0.0001. D = Day.

The effector compartment showed a distinct response compared to the lymphoreplete and ‘late’ post-alemtuzumab settings. In vehicle-treated mice, both CD8 and CD4 Teff proliferation decreased, significantly so for CD4 Teffs (Fig. 6C)—consistent with the expected slowing of lymphopaenia-induced proliferation as the lymphocyte niche refills. IL-2 promoted proliferation of CD8 Teffs and NK cells but had no effect on CD4 Teffs and Tregs. Nevertheless, lymphocyte subset frequencies did not change substantially: CD4 T cells increased slightly (not reaching significance), while NK cells decreased. These shifts were present in both IL-2 and control groups, likely as a consequence of recent alemtuzumab treatment (Supplementary Fig. 10C).

In summary, IL-2 administration early post-alemtuzumab failed to enhance Treg proliferation or frequency, despite stimulating effector cell populations. Collectively, these data suggested that despite a potentially narrower therapeutic window, Tregs can be preferentially expanded with IL-2, provided it is not administered early post-alemtuzumab. We therefore proceeded with a mechanistic experimental medicine study of IL-2, in the form of Proleukin, in people with MS 6 months or more post-alemtuzumab.

### Low-dose IL-2 is well tolerated but fails to expand Tregs in people with MS 6+ months post-alemtuzumab

Given IL-2’s potentially narrow therapeutic window post-alemtuzumab, we adopted a dose close to the low intermittent dosing regimen optimized in patients with autoimmune diabetes^25^—0.3 × 10^6^ IU/m^2^ twice weekly. Based on a re-analysis of the type 1 diabetes data,^25^ we elected to give our participants six doses, which we determined should be sufficient to expand Tregs and detect Teff expansion, if present (Supplementary Fig. 2).

Six people with MS treated with alemtuzumab ≥6 months previously were recruited from the NHS MS service at Cambridge University Hospitals in February 2021 and September 2021. Median disease duration from diagnosis was 9 years (range 1–24 years), and the mean Expanded Disability Status Scale score was 2 (0–3.5). Baseline patient characteristics are shown in Supplementary Table 3.

Blood was sampled for Treg measurement twice without IL-2 treatment (1–2 weeks apart), again just before IL-2 treatment commenced, and at the end of IL-2 treatment. Six consecutive injections of IL-2 were tolerated without any serious adverse events (Table 1). The most commonly reported adverse event was an injection site reaction, all of which were grade I and self-resolved. There were no new instances of autoimmunity and no decompensation of pre-existing autoimmunity occurred during the study (Supplementary Table 4).

**Table 1:** Safety and adverse events during experimental medicine study

Type of AE	Category	Participants
Total number	Serious AE	0
	Non-serious AEs	12
Severity	AEs by severity	
	Mild	11/12 (91.7%)
	Moderate	1/12 (8.3%)
Relatedness	Related	8/12 (66.7%)
	Unrelated/unlikely	4/12 (33.3%)
Related AEs by severity	Mild	7/8 (87.5%)
	Moderate	1/8 (12.5%)
All related AEs	Injection site reaction	4 (grade I)
	Fatigue	1 (grade I)
	Headache	1 (grade I)
	Exacerbation of MS symptoms due to heat	1 (grade I)
	Worsening of existing Graves disease	1 (grade I)
Unrelated AEs	Fatigue	1 (grade 2), onset after treatment end
	Food poisoning	1 (grade 1), prior to treatment start
	Mild neutropaenia	1 (grade 1), onset after treatment end
	Abnormal TSH	1 (grade 1), probable lab error

AE = adverse event; MS = multiple sclerosis; TSH = thyroid stimulating hormone.

The primary end point of the study- a significant percentage change in Treg frequency after six Proleukin injections, compared to change in the same patient without treatment, was not achieved (Fig. 7A). There was a 11.77% ± 24.25% change in Treg frequency after IL-2 dosing, which differed significantly from the Treg expansion previously reported in type 1 diabetes under nearly identical dosing (Supplementary Fig. 11). CD127^lo^CD25^hi^ Treg frequency remained stable throughout dosing, with a slight decline at Day 56 (4 weeks post-dosing). To account for potential concurrent changes in CD4 Tregs and CD4 Teffs, absolute cell counts were assessed. CD4 Treg absolute counts remained stable throughout the study (Fig. 7B). There were no significant changes in the frequencies of naïve or memory Tregs, Helios^+^ Tregs or CD39^+^ Tregs, previously reported to increase after IL-2^49,50^ (Supplementary Fig. 12). CD56^bright^ NK cells, which are highly sensitive to IL-2,^24,51^ increased in three of six individuals but did not reach statistical significance overall (Fig. 7C).

**Figure 7:**
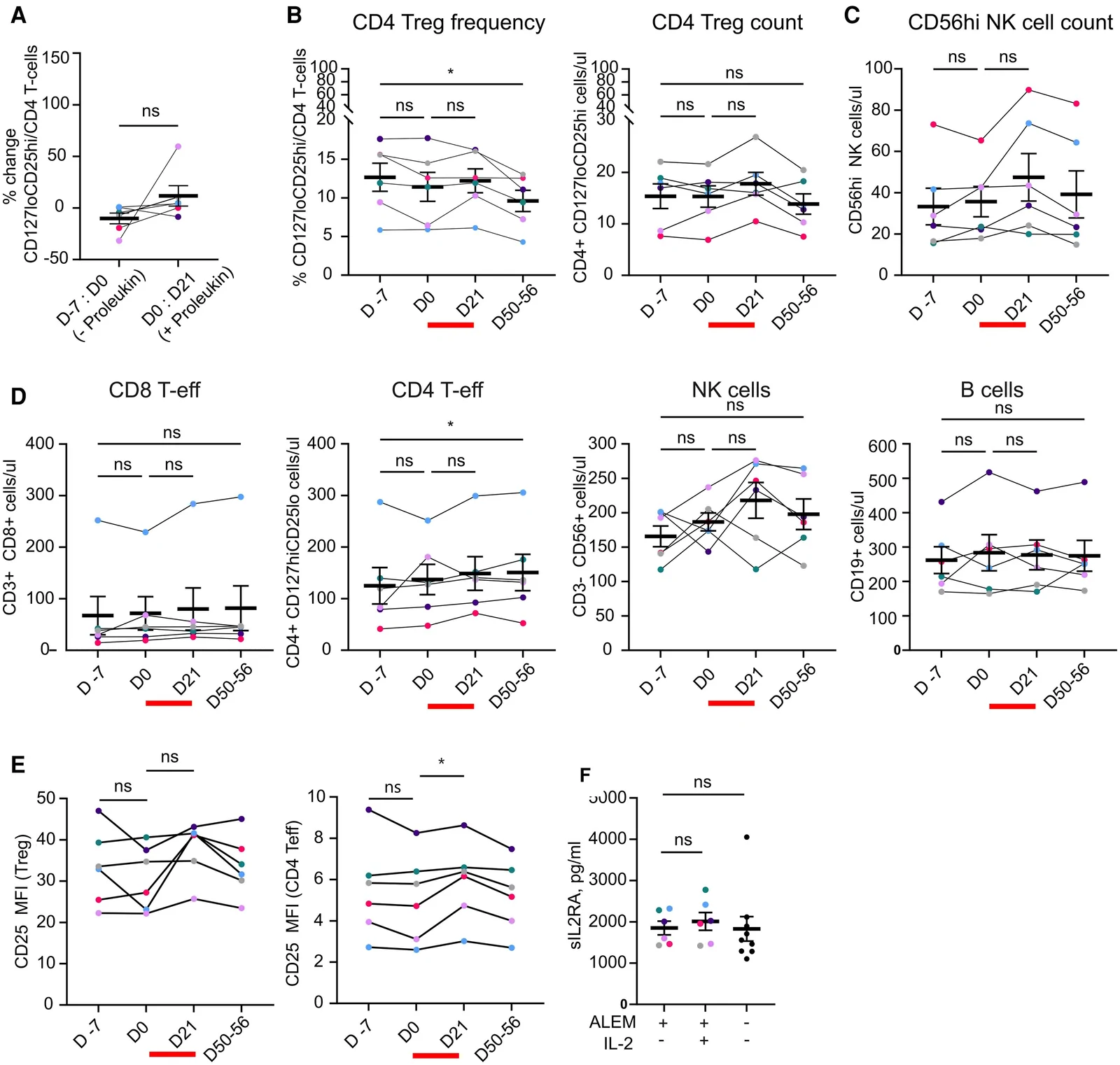
**Experimental medicine study of low-dose IL-2 post-alemtuzumab shows an unexpected lack of regulatory T cell expansion.** Participants received six subcutaneous injections over 3 weeks of IL-2 (Proleukin) at a dose previously optimized for regulatory T cell (Treg) expansion in autoimmune diabetes; cell frequencies and counts were measured in blood by flow cytometry without IL-2 (1–2 weeks apart) and again pre-/post-IL-2. (**A**) Change in Treg frequency after IL-2 treatment versus without treatment—the primary outcome—was not significantly different (ns). Wilcoxon matched-pairs signed rank test: *P* = 0.156 (two-tailed), *n* = 6 pairs. (**B**) Treg frequency and absolute Treg cell numbers did not increase during IL-2 treatment [Treg frequency: repeated measures (RM) ANOVA *F*(1.918,9.590) = 6.522, Sidak’s multiple comparisons test *P* = 0.03 for Day 56 versus Day −7, ns for all others; Treg count: RM-ANOVA *F*(2.27,11.34) = 4.34, *P* = 0.036; Sidak’s test: ns for all pairwise comparisons]. (**C**) CD56^bright^ natural killer (NK) cells, which are very sensitive to IL-2, trended up during IL-2 treatment, but their increase was not significant (RM-ANOVA with Sidak’s test, *P* = 0.087). (**D**) Absolute cell numbers of different effector lymphocyte populations remained stable; CD4 Teffs increased 4 weeks after the end of treatment, likely because of ongoing immune reconstitution. RM-ANOVA *F*(1.20,5.99) = 2.03, *P* = 0.207; Sidak’s test: Day –7 versus Days 50–56, *P* = 0.020, ns for all other pairwise comparisons. (**E**) CD25 expression transiently increased on CD4 Teffs but not Tregs during IL-2 dosing. An increase in CD25 denotes cell activation. RM-ANOVA *F*(1.814,9.071) = 3.845 (*P* = 0.065, not significant for overall effect). *Post hoc* Šídák’s test indicated a modest increase from Day 0 to Day 21 (*P* = 0.049). (**F**) sIL2RA (soluble CD25), suggested to inhibit IL-2 signalling,^48^ did not increase during IL-2 treatment, and was no different in this patient cohort compared to multiple sclerosis patients who had not received alemtuzumab first (these are the sera from nine individuals, who did not participate in the experimental medicine study and whose immunophenotyping is shown in Fig. 1). Individual participants in all plots are denoted by the same colour, connected by a line. Red bars under the plots denote the treatment period with IL-2. *P < 0.05. ALEM = alemtuzumab.

Effector T-cell, NK-cell and B-cell counts were also unchanged during IL-2 dosing (Fig. 7D). However, CD4 Teff counts were higher at Day 56 than before treatment, explaining the slight reduction in Treg frequency over the same period. Without a matched ‘no IL-2’ control group, it is not possible to determine whether IL-2 had influenced the rate of CD4 Teff reconstitution. However, over the study's short duration, CD4 Teffs appeared to repopulate at a rate similar to previous reports.^32^

CD25 expression on Tregs is expected to increase within 1 day of a single s.c. IL-2 dose and to remain easily detectable for approximately 3 days.^24^ In our study, CD25 expression on Tregs did not increase post-IL-2 treatment, but instead a transient rise was observed on CD4 Teffs (Fig. 7E), indicating activation. This did not, however, correlate with a higher absolute CD4 Teff count at the final visit (data not shown).

Serum sIL2RA (sCD25), another marker of lymphocyte activation that typically rises with ldIL2 treatment, did not increase (Fig. 7E). Furthermore, sIL2RA levels were comparable to people with MS who had not received alemtuzumab previously (individuals shown in Fig. 1D and E), and fell within the range reported for healthy controls.^48^ Therefore, while sIL2RA has been reported to neutralize IL-2,^48^ this did not explain the lack of Treg expansion observed in our study.

## Discussion

Despite its high efficacy, alemtuzumab use in MS is limited by its frequent association with secondary autoimmune diseases arising during immune reconstitution, with no predictive markers or mitigation strategies available. Given the critical role of Tregs in immune tolerance, we investigated whether low-dose IL-2, an established method for preferentially expanding polyclonal Tregs, which has been associated with clinical improvement in other inflammatory settings, could be applied post-alemtuzumab. A key concern was whether IL-2 would still favour Treg expansion, given the altered immune landscape.

We demonstrated that during immune reconstitution post-alemtuzumab, naïve CD4 Teffs acquire high-affinity IL-2 receptors, likely due to elevated IL-7 levels,^52,53^ and as a consequence become more responsive to IL-2 *in vitro.* IL-7 is known to drive the proliferation of healthy CD4 naïve T-cells, helping to maintain repertoire diversity despite age-related declines in thymic output,^14^ and in lymphopaenic settings, such as post-chemotherapy, it reciprocally regulates CD127 and CD25 expression^34^ through multiple mechanisms^54^—paralleling our observations.

Given the risk that IL-2 could also activate non-Tregs, we first assessed its effects *in vivo* using human CD52-expressing mice treated with alemtuzumab. Here, ‘late’ IL-2 administration induced Treg expansion, whereas ‘early’ IL-2 delivery failed to drive Treg proliferation and instead activated non-Tregs. Consequently, we proceeded cautiously with a proof-of-concept experimental medicine study in people with MS who had received alemtuzumab at least 6 months prior, using an IL-2 dose (0.3 × 10^6^ IU/m^2^ twice weekly) close to the dose optimized for Treg expansion in autoimmune diabetes (0.26 × 10^6^ IU/m^2^ every 3 days).^25^ This is lower than doses used in other autoimmune diseases, which typically involve ∼1 × 10^6^ IU/day and/or an initial period of daily dosing^26^ to promote rapid Treg expansion.

The intervention was safe and well-tolerated. No signal of altered autoimmune risk was observed; however, this was entirely expected as the study was not designed, powered or of sufficient duration to assess clinical outcomes.

Despite predicting a 46% increase in Tregs based on the autoimmune diabetes literature,^25^ we did not observe clear or consistent Treg expansion. On the advice ot the Trial Steering Committee, the study was terminated early (six rather than seven participants dosed), on the basis of futility. While very subtle shifts in Treg frequency cannot be definitively excluded in a cohort of this size, the absence of a biologically meaningful increase represents a clear deviation from the predicted response. Several factors may explain this unexpected result.

First, the IL-2 dose may have been insufficient. This is unlikely, provided that MS Tregs are as responsive to IL-2 as those from patients with autoimmune diabetes or healthy controls.^55^ In the reference type 1 diabetes study, using a near-identical regimen, Tregs expanded in all patients (mean 46%), with 11 of 12 increasing by >20% after six doses^25^ (Supplementary Fig. 11). Even lower IL-2 doses have been shown to significantly expand Tregs in healthy volunteers.^55^ In contrast, only one of six alemtuzumab-treated MS patients in our study showed a transient >20% increase in Treg frequency. Studies across a wide range of non-MS autoimmune diseases^20,23,56^ have reported ∼70% Treg expansions with higher and more frequent IL-2 dosing. However, our *in vitro* data indicate that higher IL-2 doses post-alemtuzumab are not feasible, as they carry a substantial risk of activating T-effectors.

Second, IL-2 may have been administered too early post-alemtuzumab. In mice, early IL-2 dosing failed to expand Tregs as a proportion of CD4 T-cells. Absolute cell numbers were not measured, but we observed no increase in Treg Ki-67, indicating a true failure of IL-2 to induce Treg expansion, rather than a detection issue caused by other populations shifting. The half-life of alemtuzumab in mice is unknown, so it is possible that at the early timepoint, alemtuzumab continued to deplete expanding Tregs- but this would not explain the absence of proliferation. More plausibly, Tregs early post-alemtuzumab may already be maximally proliferative and unable to further respond. Our human spectral immunophenotyping data support this hypothesis, as we observed increased Treg proliferation for 6 months post-alemtuzumab. Moreover, outside the MS context, when alemtuzumab is used for lymphodepletion prior to haematopoietic stem cell transplantation, early IL-2 administration similarly fails to expand Tregs.^57^ However, in our clinical study, all but one participant received IL-2 at least 9 months post-alemtuzumab, a period when Treg frequencies stabilize,^4^ making suboptimal timing an unlikely explanation.

Third, Tregs in our patient cohort may have been functionally exhausted- a dysfunctional state induced by chronic stimulation, characterized by a distinct transcriptional profile, impaired signalling, reduced cytokine production, and limited proliferation.^58^ Lymphopaenia-driven homeostatic proliferation requires sustained TCR signalling,^59^ and proliferative senescence markers (CD57, PD-1) increase in bulk T cells post-alemtuzumab.^4^ Tonic TCR signalling can likewise induce Treg exhaustion, marked by elevated PD-1 and reduced proliferative capacity.^60^ In mouse Tregs, PD-1 limits IL-2-driven proliferation, though in its absence, unchecked proliferation results in apoptosis and no net expansion^61^ Our human spectral immunophenotyping data revealed persistently high PD-1 expression in memory Tregs beyond 6 months post-alemtuzumab, potentially restricting Treg proliferation. Whether the observed lack of Treg expansion reflects impaired proliferation, survival failure, or another intrinsic Treg dysfunction post-alemtuzumab remains unclear. Factors extrinsic to Tregs may also influence their expansion. For example, other cytokines can alter the threshold for IL-2-driven Treg proliferation.^62^ We also considered whether the increased proportion of CD25^+^ naïve Teffs post-alemtuzumab might act as an IL-2 sink. While plausible, our data suggest this is unlikely because Tregs retained high sensitivity to IL-2 as assessed by pSTAT5 levels after *in vitro* stimulation, and healthy Tregs are capable of maintaining proliferation at even minimal STAT5 phosphorylation.^17^ Finally, the absence of Treg expansion may not be alemtuzumab-specific. Our open-label mechanistic study did not include a non-alemtuzumab control group, so we are unable to definitively address whether the lack of Treg expansion reflects an intrinsic MS-related defect or perturbations arising during immune reconstitution. In our mouse model, Treg expansion was higher in mice not pre-treated with alemtuzumab (a 1.43-fold increase versus a 1.34-fold increase in the ‘late’ alemtuzumab setting), but this difference was not statistically significant, with the caveat that the groups were small. Formal testing of biological equivalence would require an impractically large number of mice (>60/condition). Therefore, whether alemtuzumab attenuates IL-2 driven Treg responses in humans remains unresolved.

Interestingly, a recent placebo-controlled trial of ldIL2 in people with MS who had not been treated with alemtuzumab and who received a higher IL-2 dose over several months, also demonstrated delayed and reduced Treg expansion (20% observed versus 200% predicted).^22^ This raises the broader question of whether MS Tregs exhibit a generalized hypo-responsiveness to IL-2, their key homeostatic cytokine.

Associations of *IL2RA*, *IL2* and other IL-2 pathway genes with MS highlight the central role of IL-2 in immune homeostasis and Treg function^63^ but a direct link between risk variants and Treg dysfunction is limited. *IL2RA* risk variant rs11256593 has been associated with reduced CD25 expression on a small subset of Tregs, without assessing the impact on IL-2 responsiveness.^64^ Another *IL2RA* risk variant, rs2104286,^65^ has been linked to reduced IL-2–induced STAT5 phosphorylation in CD4^+^CD25^hi^ cells, but this broad Treg definition, and our later recognition that this SNP is tagging, not causal,^66^ are a significant limitation. A comparable IL-2 signalling defect was not observed in the Klatzmann MS IL-2 trial,^22^ and evidence for impaired signal transduction and Treg proliferation in untreated MS is currently limited to one report under particular *in vitro* conditions.^67^ Signals through the TCR and CD28 also support *in vivo* Treg proliferation,^68^ modulated by IL-2,^69^ so the contribution of defects in these pathways remain to be considered.

Our findings apply specifically to recombinant human IL-2. IL-2:anti-IL2 antibody complexes, and engineered IL-2 muteins (reviewed by Raber *et al*.^70^) biased towards Treg activation may permit more sustained signalling with reduced off-target effects. Nevertheless, their selectivity still depends on the differential expression of high-affinity IL-2 receptors, which is altered post-alemtuzumab. Given that IL-2 forms the basis of most Treg therapeutic expansion strategies, and that Tregs remain a promising therapeutic target in MS—particularly as an add-on to moderate-efficacy therapies to manage breakthrough disease—Treg hyporesponsiveness is an important avenue for future investigation.

In conclusion, our data show there is no rationale for using low-dose IL-2 alone as a strategy for preventing autoimmune complications following alemtuzumab treatment. Importantly, our work also raises the possibility of intrinsic Treg IL-2 dysfunction in MS. Addressing these intrinsic defects could advance our understanding of MS pathogenesis and inform the development of more effective immunoregulatory therapies, including IL-2 muteins and combination approaches targeting additional molecular pathways.

## Supplementary Material

awag171_Supplementary_Data

## Data Availability

The data that support the findings of this study are available from the corresponding author, upon reasonable request.
