# Supplementary material for: Low-dose IL-2 fails to expand Tregs after alemtuzumab: insight into impaired immune tolerance in multiple sclerosis
Source: Brain. 2026 May 13;149(11):4035–51. doi: 10.1093/brain/awag171 (PMC13730587; doi:10.1093/brain/awag171)
Supplement: Supplementary file 1 [file awag171_supplementary_data.zip › brain-2025-03236-File010.pdf]

Supplementary Figure 1: Gating for clinical flow cytometry

(A) Modified Beckton Dickinson TBNK reagent Trucount™ tube

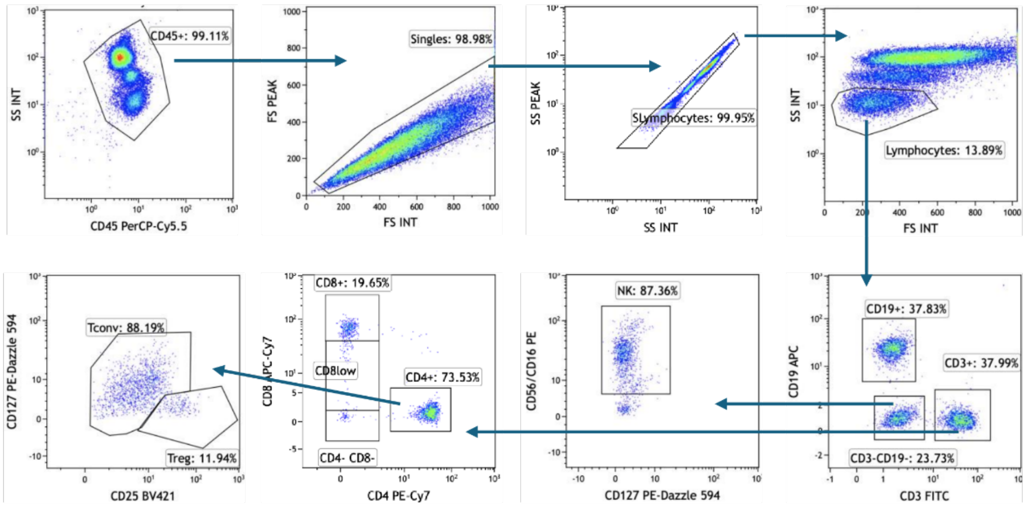

(B) Modified Beckman Coulter DURAClone Treg tube

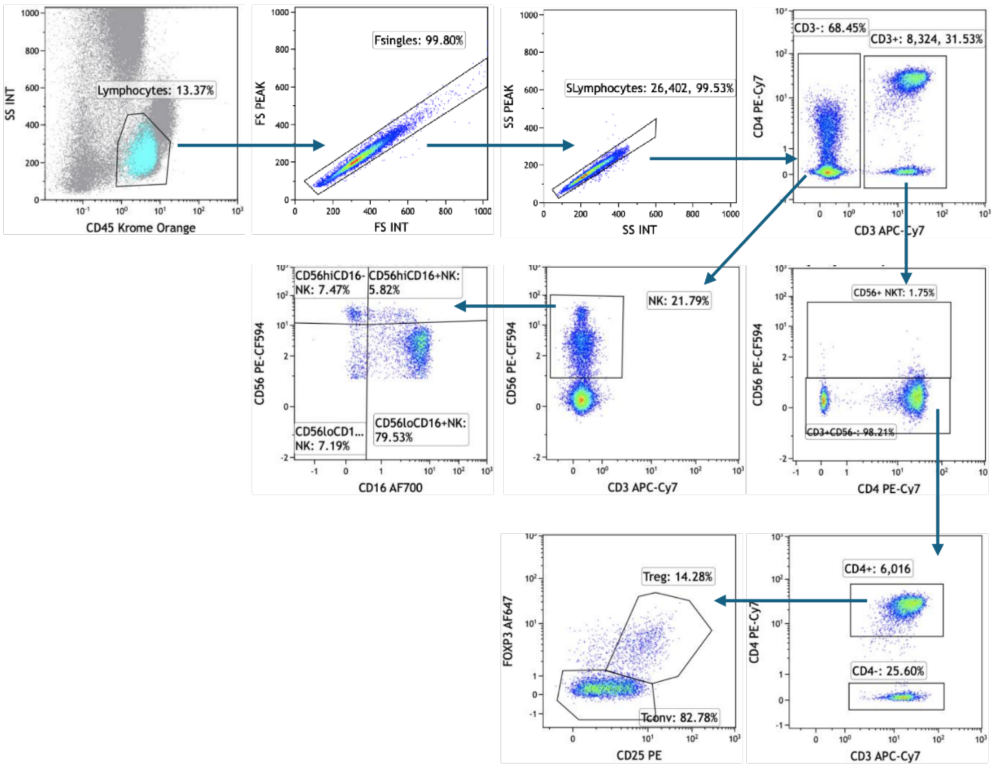

(A) Surface staining of whole blood from participants in the experimental medicine study for absolute cell counting.

(B) (B) Intracellular staining of the same samples was used to derive frequencies of FOXP3+ cells, and CD56hi NK cells.

Supplementary Figure 2: Calculations used to select the dose of Proleukin used in our human experimental medicine study in RRMS. Raw data from the type 1 diabetes dose-finding trial (Seelig et al, JCI Insight 2018) were used to calculate the response after 6 IL-2 injections and 8 IL-2 injections.

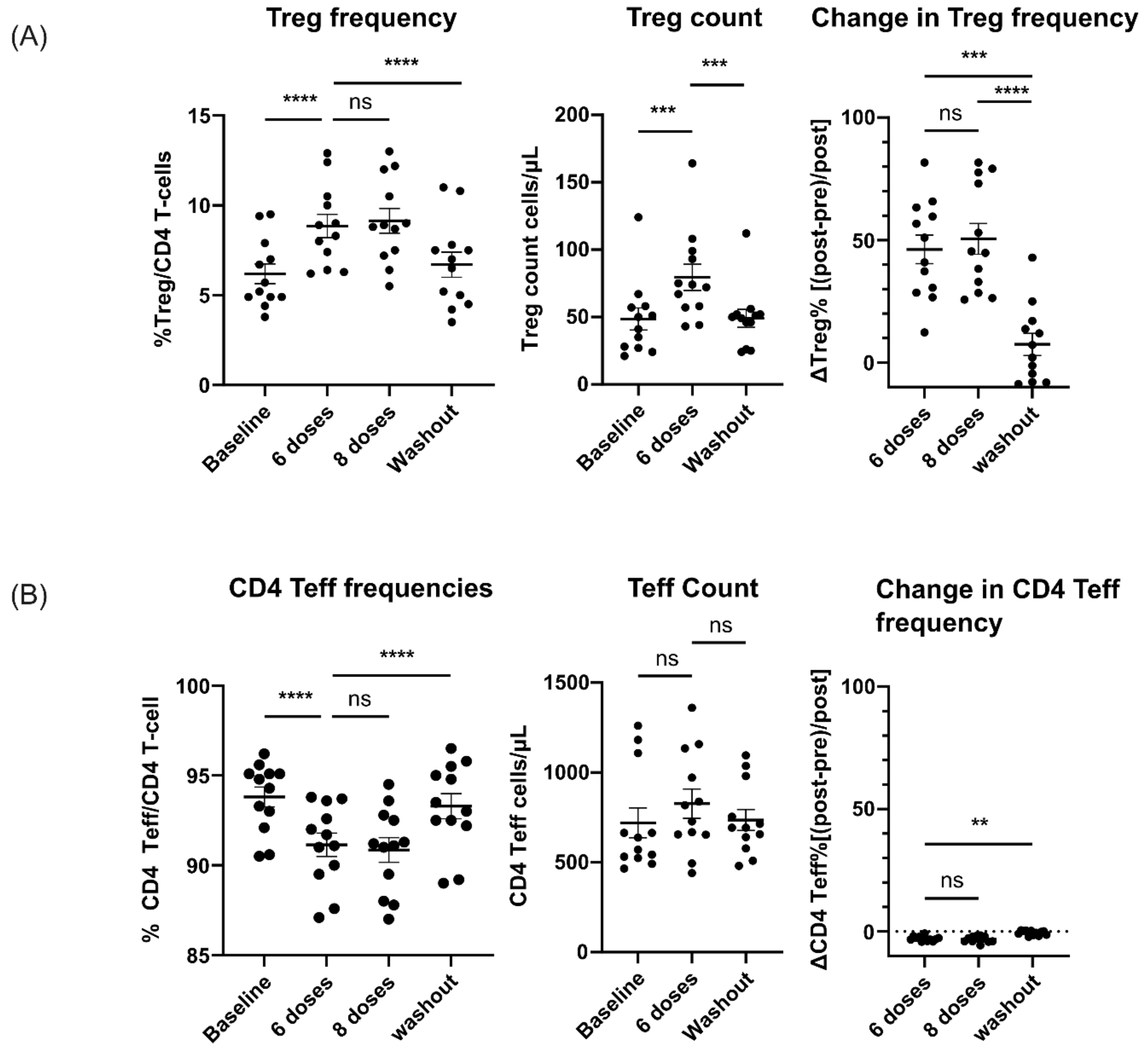

(A) Treg frequency (RM ANOVA F (2.266, 24.92) = 64.48) and absolute Treg cell count (F (1.564, 17.21) = 41.90) were both found to increase significantly after just 6 injections of IL-2, Sidak's test baseline vs 6 doses p<0.0001 for both. The primary outcome of our study was planned to be a percentage change in Treg frequency. There was no significant difference in this parameter when comparing 6 and 8 injections of IL-2.

(B) An increase in CD4 Teff count after 6 doses of IL-2 was not statistically significant. However, there was a clear trend towards an increase (p=0.053, RM ANOVA with Sidak's post-hoc test), detectable in 8 of 12 individuals. Based on our pre-clinical data, we anticipated that this effect, if present, would be exaggerated post-alemtuzumab. As we did not wish to power the study to measure a Teff increase, we judged this trend to be a sufficient signal.

Supplementary Figure 3: Sort strategy for isolating Tregs to measure TSDR demethylation

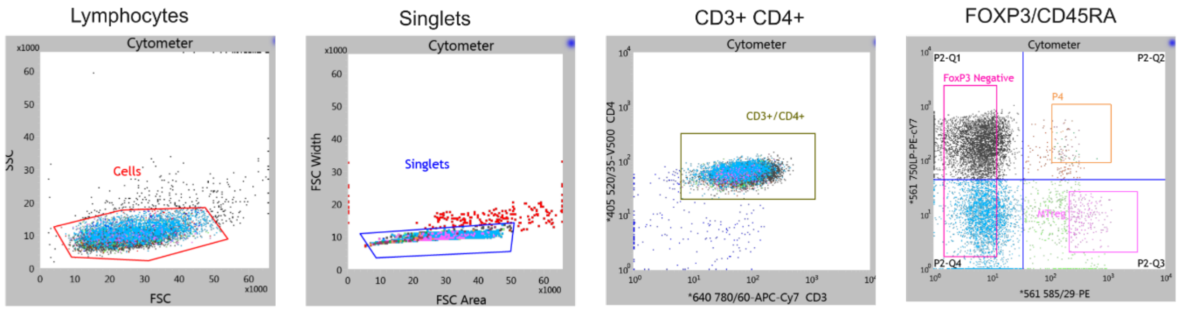

Memory Tregs for this experiment were defined using the Miyara et al convention (CD45RA-FOXP3hi CD4 T-cells) because we wished to test whether the FOXP3 expressing cells post-alemtuzumab are truly Tregs rather than activated T-effectors. The effector population in this experiment was a mix of FOXP3- naïve and memory CD4 T-cells.

Supplementary Figure 4: Sort strategy for isolating Tregs for gene expression analysis by Nanostring.

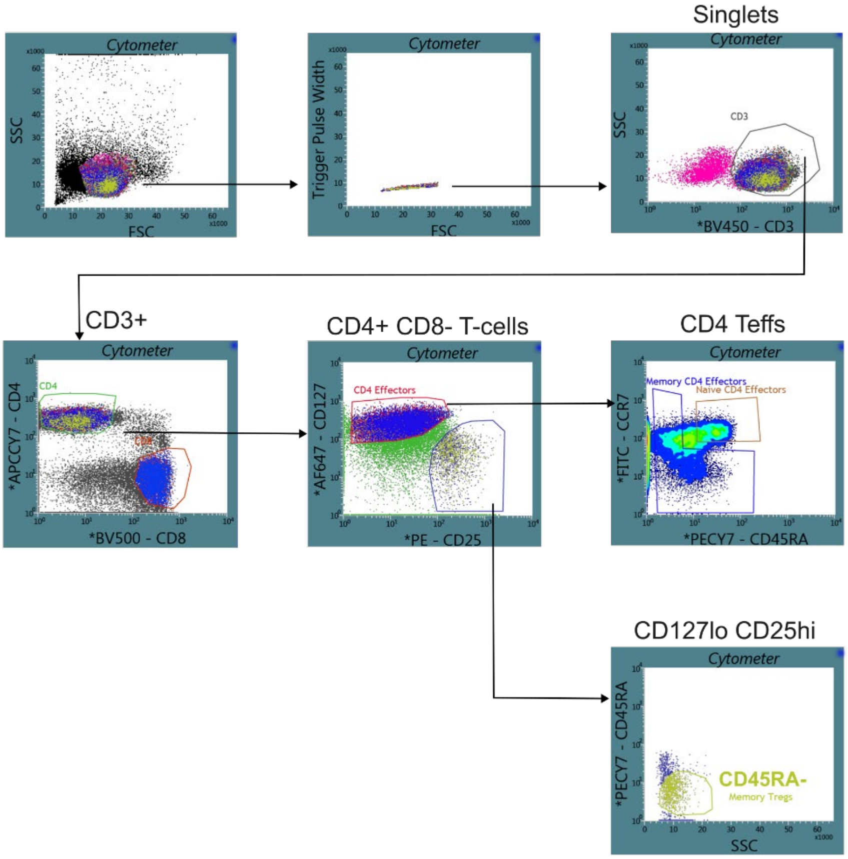

For gene expression analysis, memory Tregs were defined based on surface staining. Intracellular staining requires fixation, which can compromise RNA quality.

A

Supplementary Figure 5: Additional Treg phenotyping data.

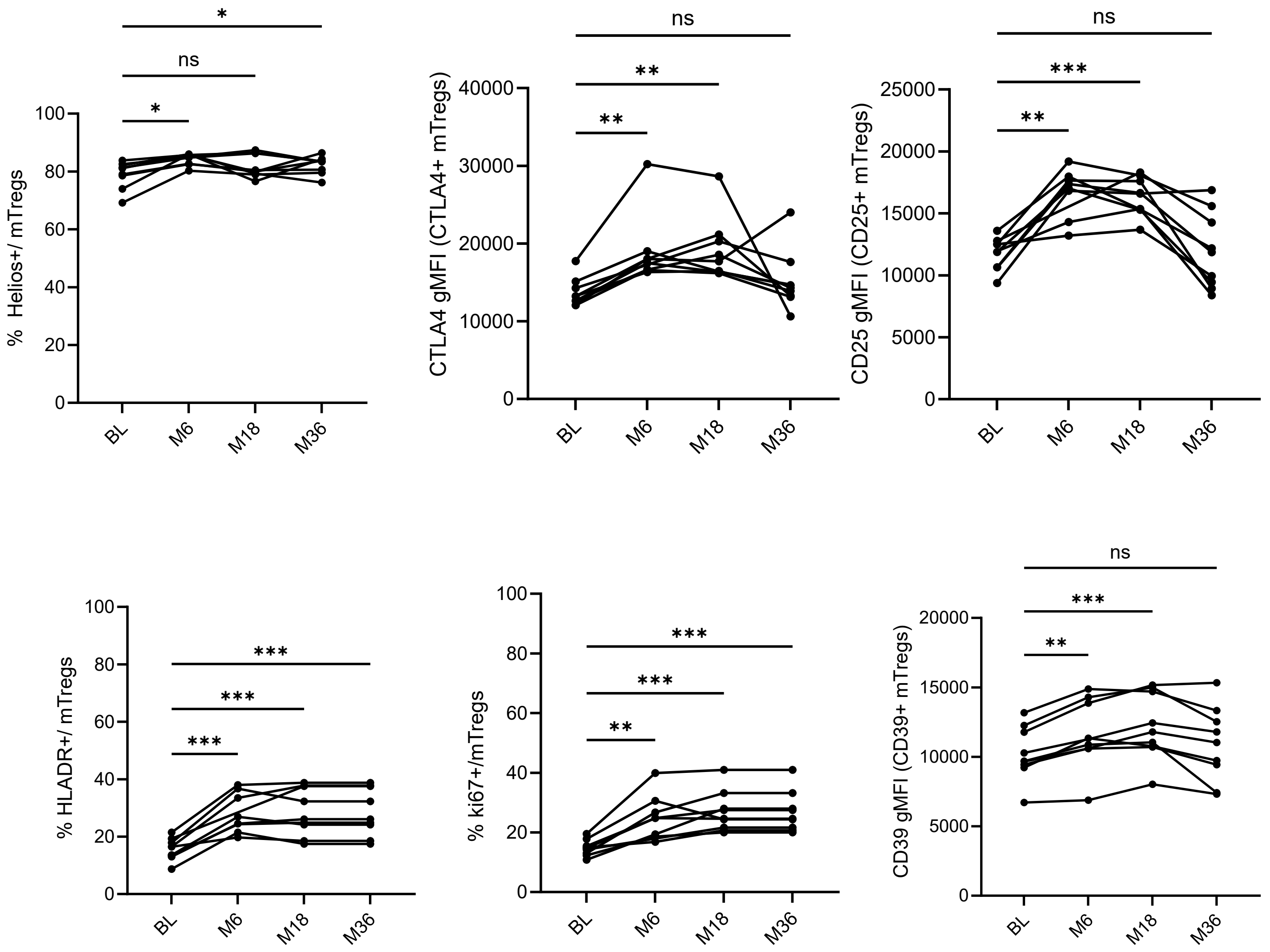

The same individuals presented in Figure 1E are shown in this figure. (A) Expression of canonical Treg markers on memory Tregs (CD127lo FOXP3hi cells), by individual donor. Corresponding to representative histograms in Figure 1E.

On the next page-

(B) CD49d increases post-alemtuzumab; it has previously been associated with reduced suppressive function particularly against memory Teffs. (C) CD226 increases post-alemtuzumab while TIGIT does not. TIGIT and CD226 compete for the same receptor, so CD226 opposes suppressive function mediated via TIGIT. (D) PD-1 increases post-alemtuzumab; it has been linked with defective Treg function in the context of glioma, though its role in Treg proliferation, survival and function is complex.

(B)

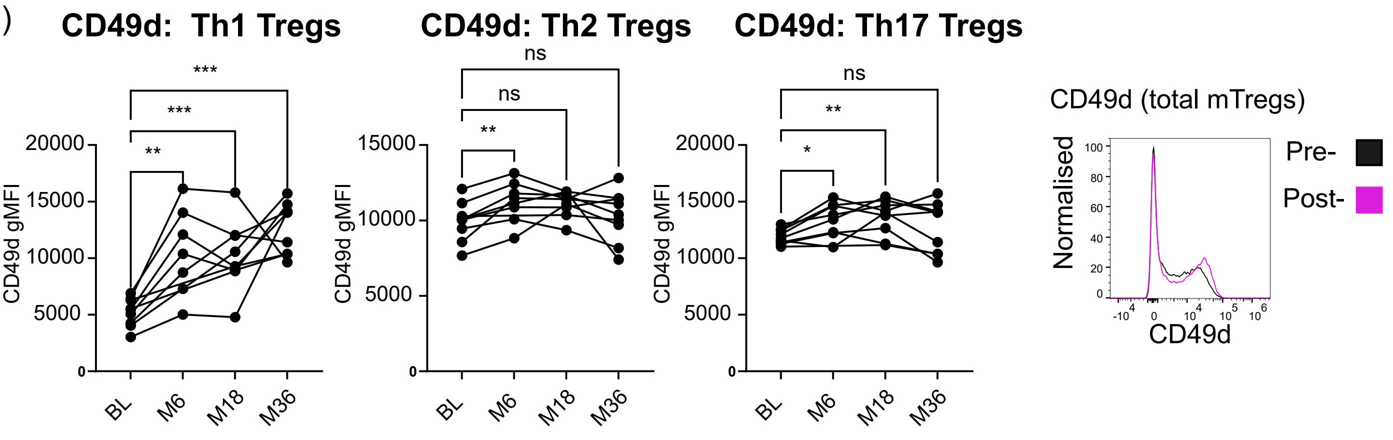

CD49d: Th1 Tregs
CD49d: Th2 Tregs
CD49d: Th17 Tregs
CD49d gMFI
BL
M6
M18
M36
***
**
*
ns
CD49d (total mTregs)
Normalised
CD49d
Pre-
Post-

(C)

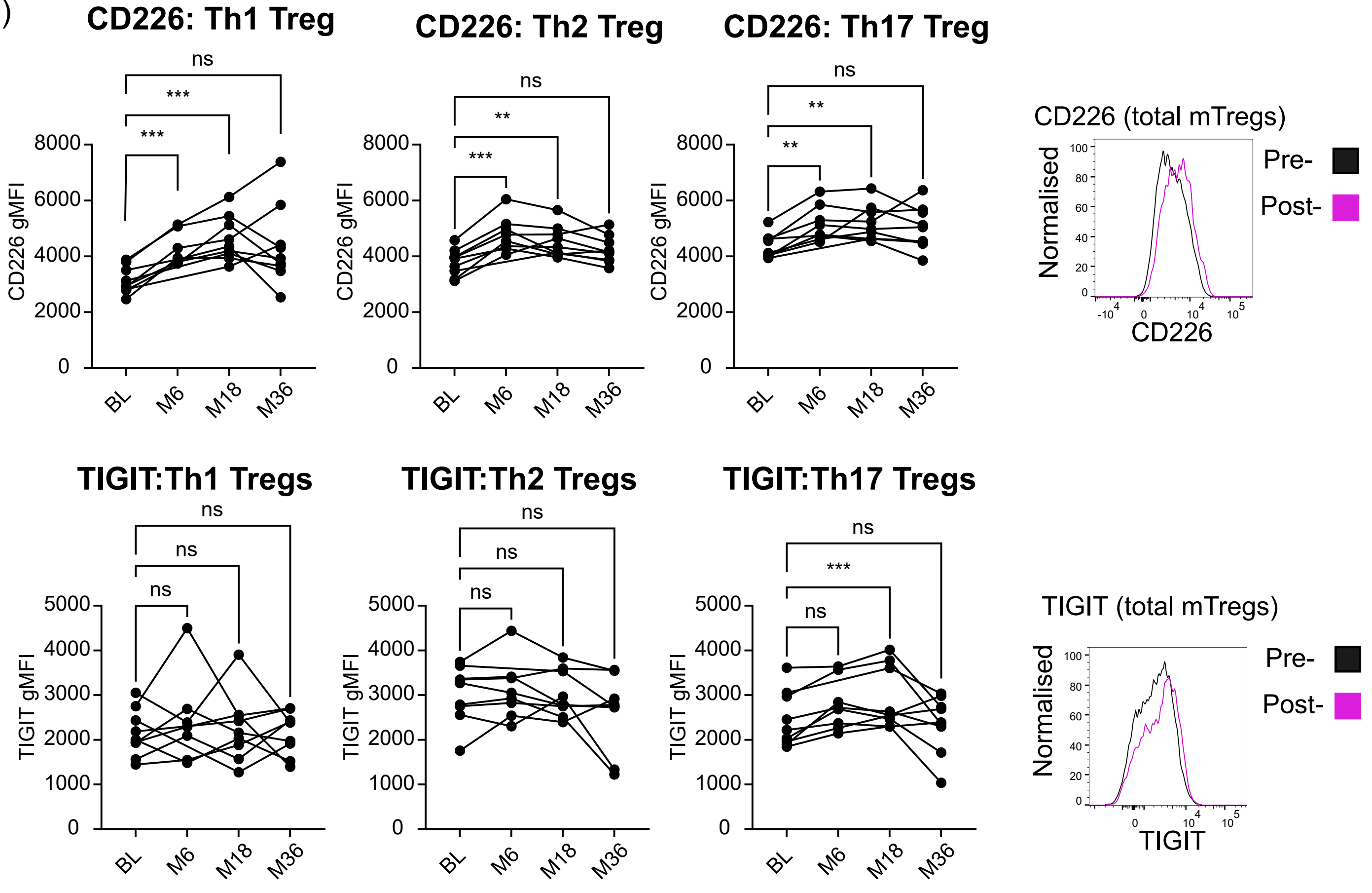

CD226: Th1 Treg
CD226: Th2 Treg
CD226: Th17 Treg
CD226 gMFI
CD226 (total mTregs)
Normalised
CD226
Pre-
Post-
TIGIT:Th1 Tregs
TIGIT:Th2 Tregs
TIGIT:Th17 Tregs
TIGIT gMFI
TIGIT (total mTregs)
Normalised
TIGIT
Pre-
Post-
BL
M6
M18
M36
ns
**
***

(D)

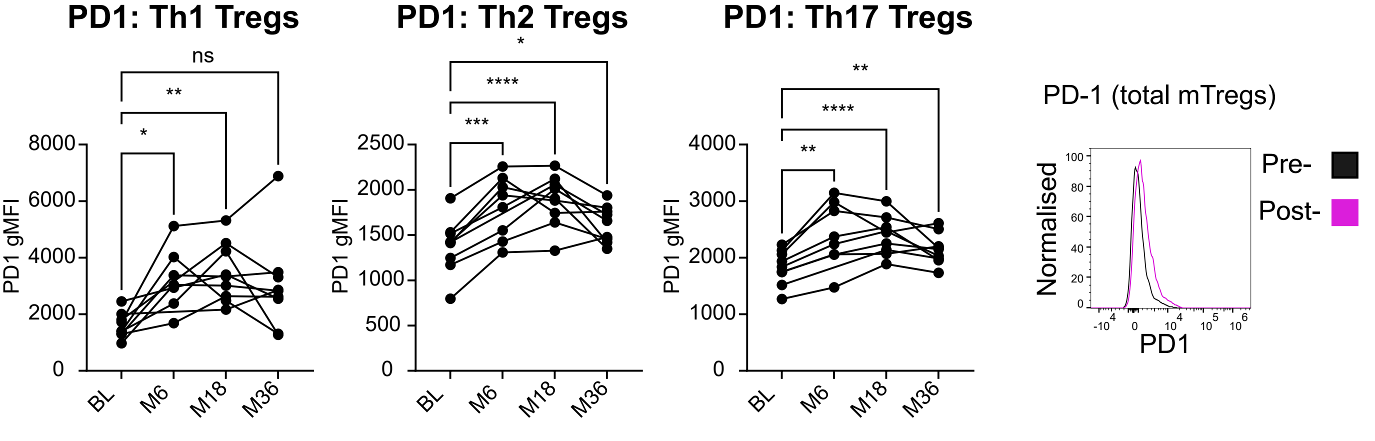

PD1: Th1 Tregs
PD1: Th2 Tregs
PD1: Th17 Tregs
PD1 gMFI
BL
M6
M18
M36
ns
*
**
***
****
PD-1 (total mTregs)
Normalised
PD1
Pre-
Post-

Supplementary Figure 6: Gating strategy for identifying lymphocyte populations for quantitative flow cytometry to measure CD25 and CD122 expression

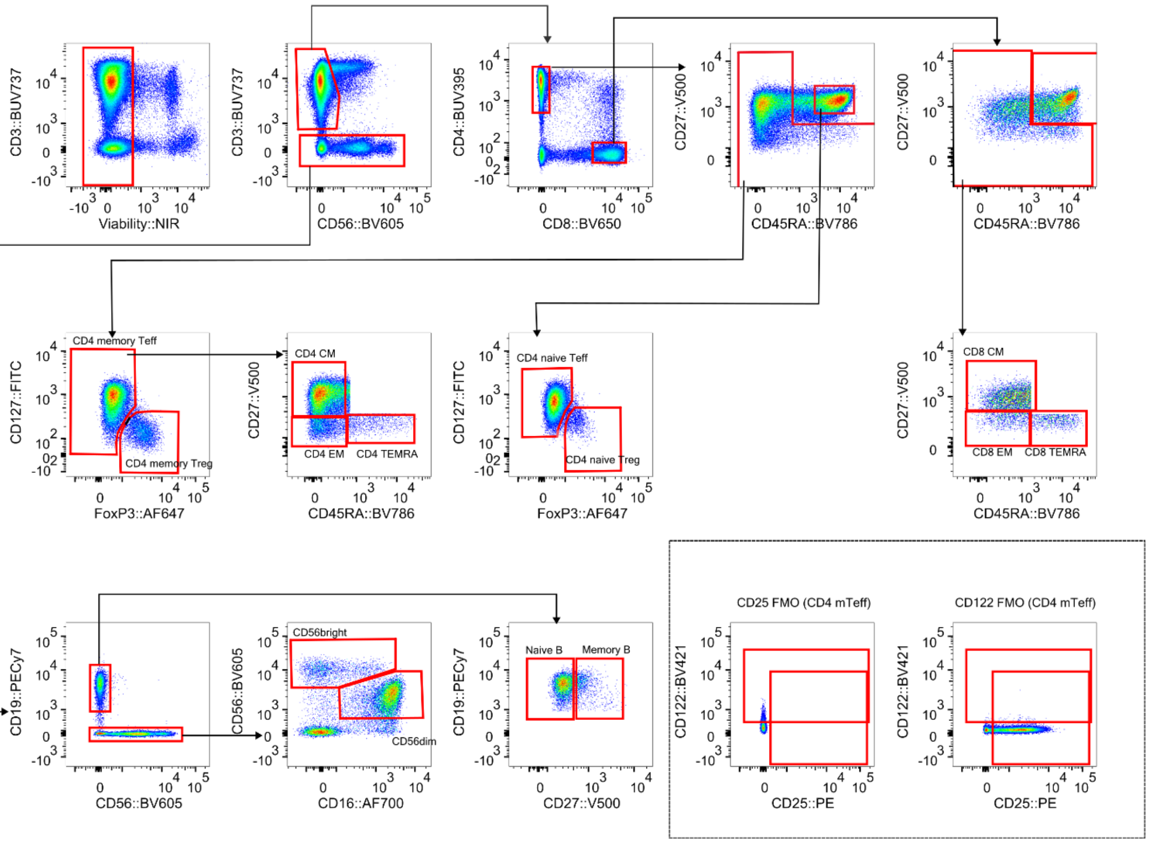

CD25 and CD122 gates have been set using FMO controls (inset).

Supplementary Figure 7: Additional quantitative cytometry data for CD25 and CD122 expression post-alemtuzumab

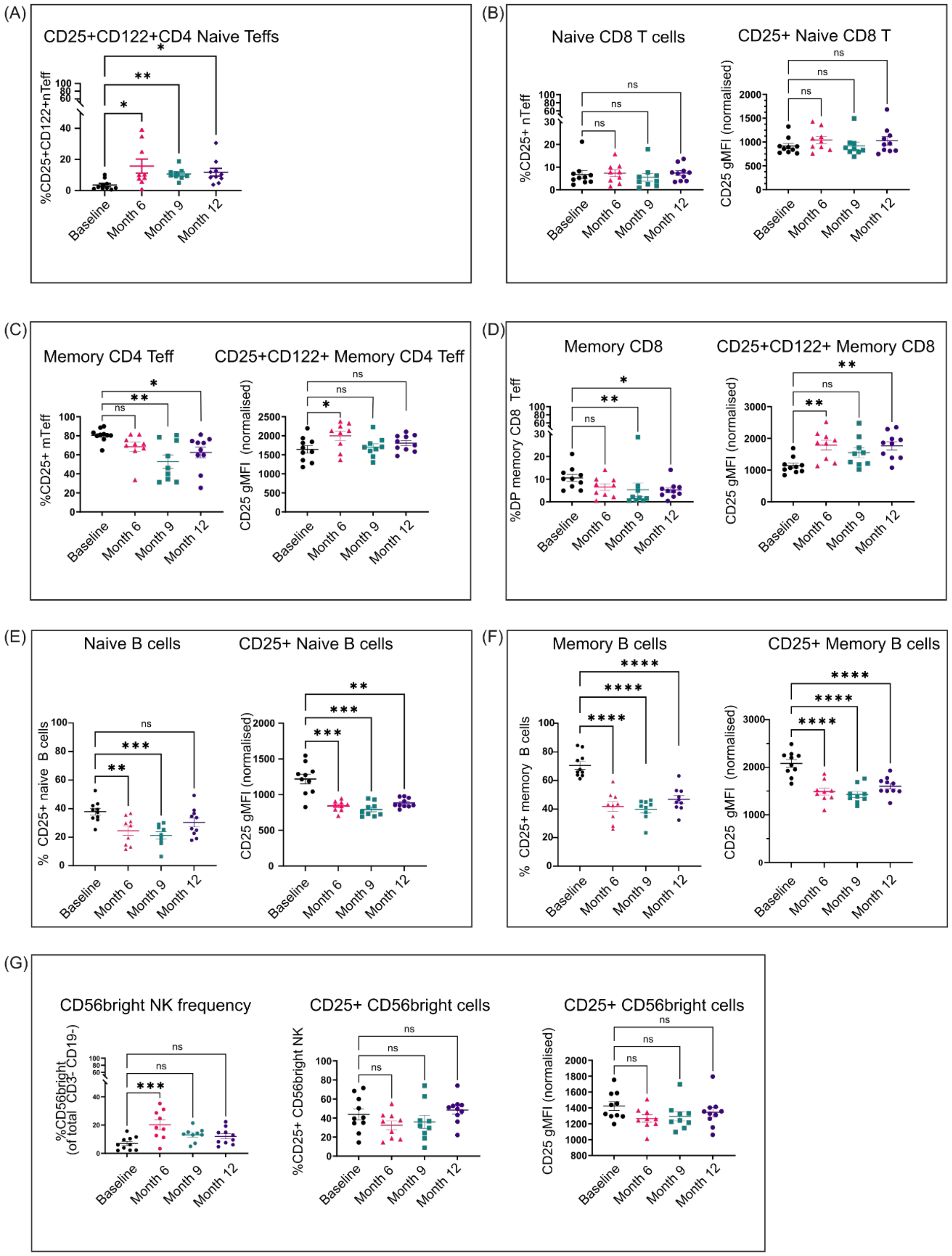

(A) Frequency of CD25+ CD122+ naïve Teffs. (B) Frequency of CD25+ naïve CD8 Teffs, and CD25 expression on them. (C) CD25+ Memory CD4 Teffs and CD25 expression on them. (D) CD25+ CD122+ memory CD8 Teffs, and CD25 expression on this subset.

(E) Naïve B cells and CD25 expression on them, and (F) Memory B-cells and CD25 expression on them. This decrease is expected, since immune reconstitution results in less activated, less mature B-cells repopulating from bone marrow. (G) CD56^{bright} NK cells are enriched at 6 months but do not change their expression of CD25 (they are universally positive for CD122, not shown).

Supplementary Figure 8: Unsupervised and semi-supervised dimensionality reduction analysis of CD25 and CD122 expression.

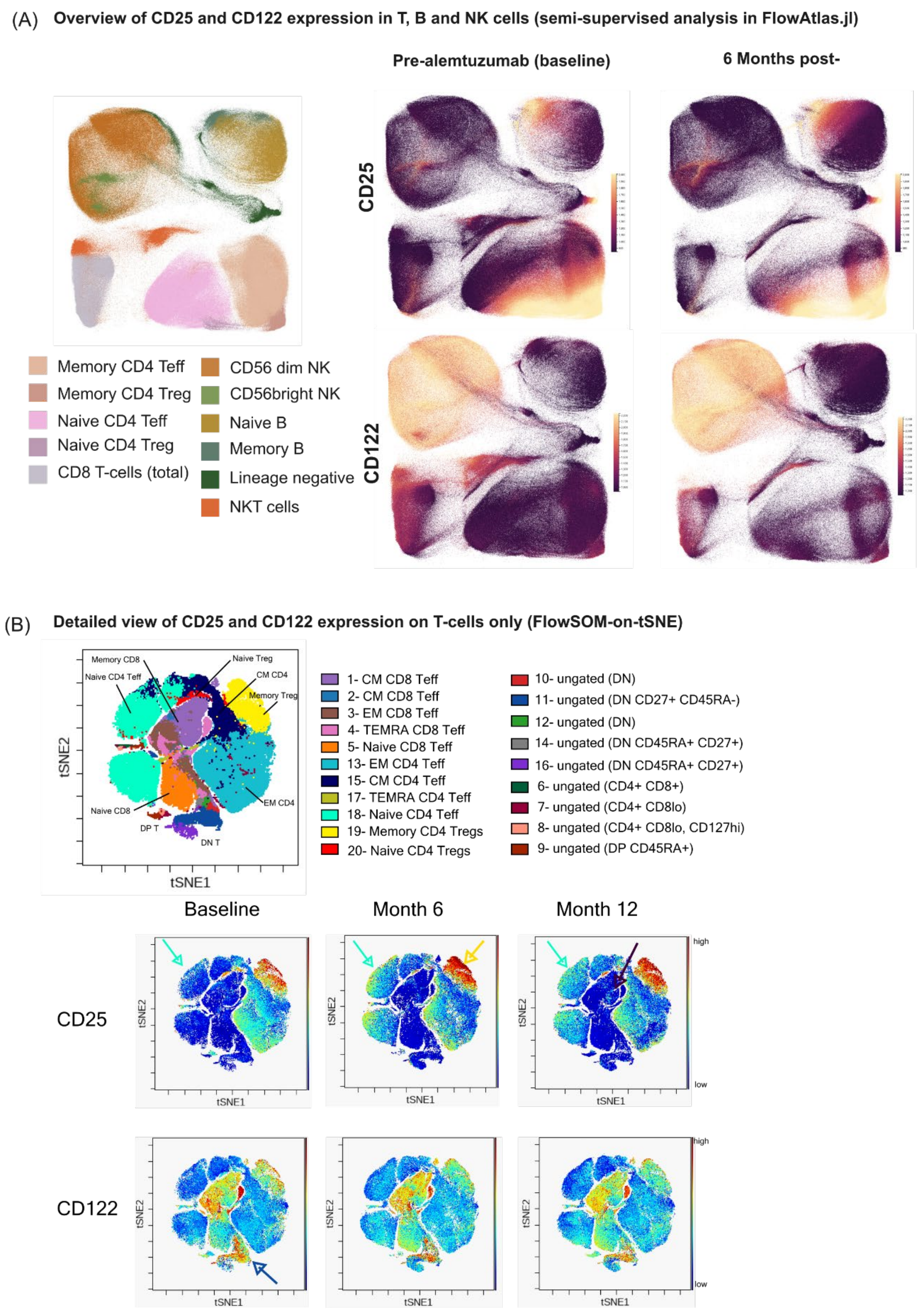

(A) Semi-supervised analysis using FlowAtlas.jl. FlowAtlas is an analysis tool which leverages dimensionality reduction and clustering using the EmbedSOM algorithm. Its key advantage is the ability to process very large datasets quickly, and without specialist computing equipment, down sampling, or concatenating samples. Initial large populations are defined by the user in FlowJo, then explored on a map interface where each subpopulation can be

magnified and explored in detail. The above heatmaps are coloured by cell type, CD25 and CD122 expression respectively. FlowAtlas.jl can be downloaded here: https://github.com/gszep/FlowAtlas.jl and a more detailed description of its use is found in reference 46 Georgieva, Coppard, Szep et al. *Frontiers in Immunol* (2024)

(B) Unsupervised analysis in FlowSOM (as implemented in Cytobank v9). Live single T-cell events have been exported and concatenated, grouped by time point. The FlowSOM-on-viSNE map is automatically generated (settings for FlowSOM: hierarchical consensus clustering, 20 metaclusters, 144 clusters, seed 1127666712, clustering channels: all, excluding CD25 and CD122; down-sampling= yes, equal N= 100,000 events/file, normalisation = none). The resulting clusters were identified manually by inspecting expression level heatmaps of lineage markers. CD25 and CD122 expression overlayed on the FlowSOM-on-viSNE map provide an overview of any unexpected changes that may be missed in a hypothesis driven classical analysis. The arrows on the map show changes we have already discussed in the main figures and Supplementary Figure 7 (cyan= CD25 in naïve CD4 Teffs; dark blue= CD25+ memory CD8 Teffs; mid-blue= CD4- CD8- T-cells, yellow= memory Tregs). No additional major unexpected changes were seen.

Supplementary Figure 9: Individual donor responses to IL-2 *in vitro*

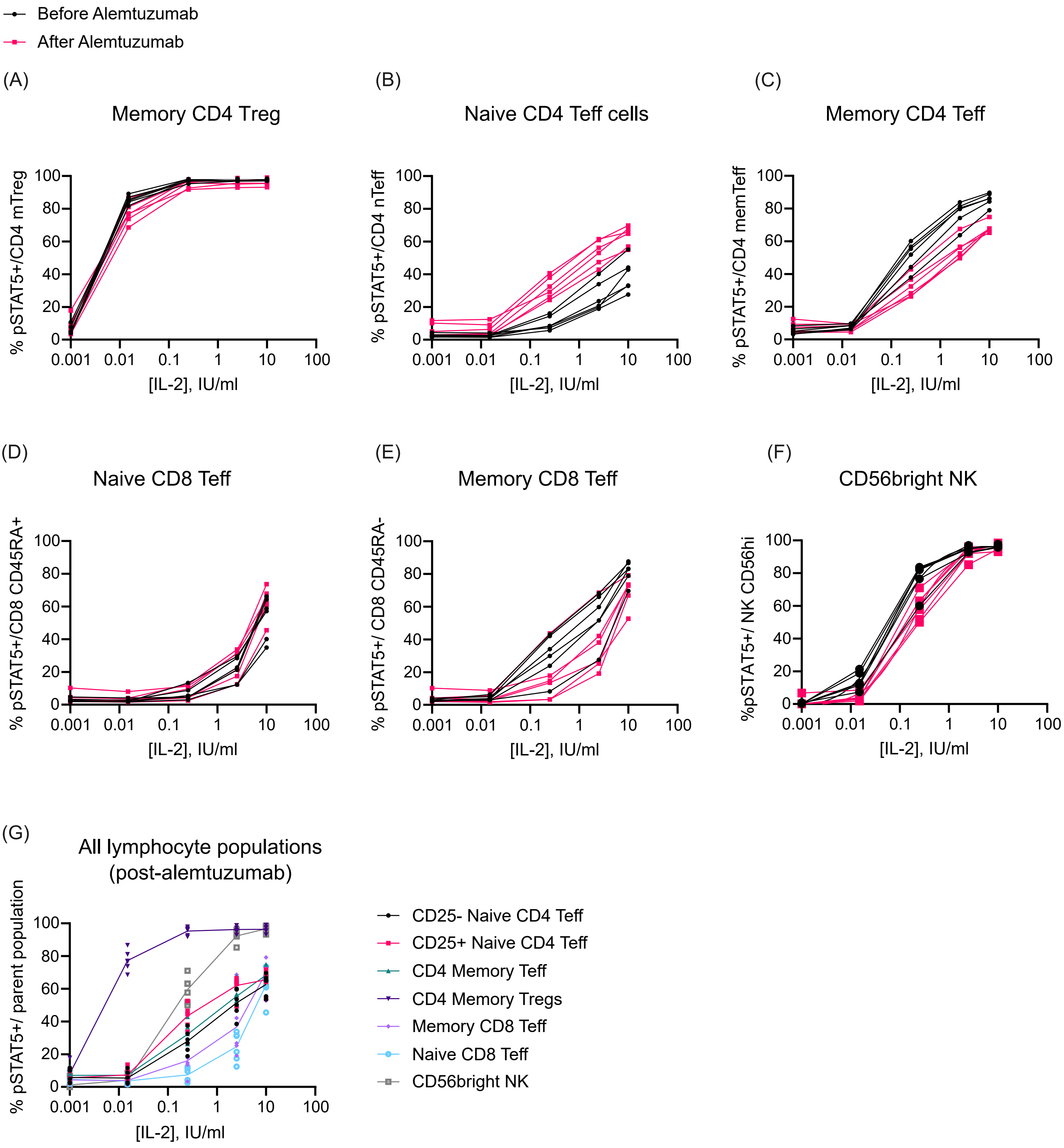

A-F: Proportion of pSTAT5+ cells within the indicated parent population pre- and 6 months post alemtuzumab treatment. Black= pre; fuschia = post. These data correspond to Figure 3. G: Comparison of pSTAT5 response post-alemtuzumab between the indicated cell populations. Lines join the means at each IL-2 concentration. Tregs remain more responsive than CD25+ naive Teffs.

Supplementary Figure 10: Additional analysis of CD52 transgenic mice dosed with IL-2 with and without prior lympho-depletion

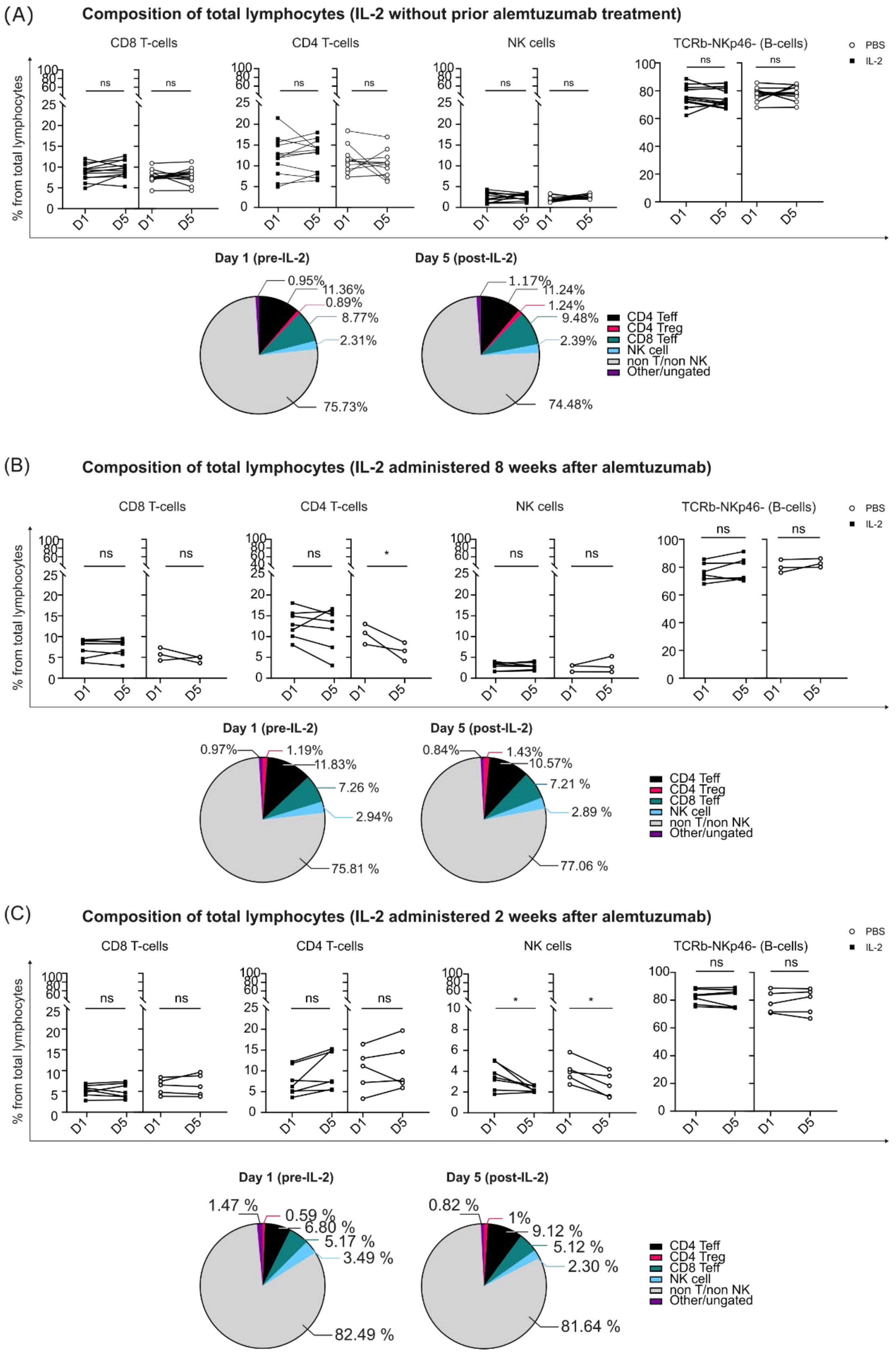

Although IL-2 causes effector cell proliferation (see Figures 5 and 6), the frequencies of major lymphocyte subsets (CD8 T-cells, CD4 T-cells, NK and B-cells) do not change significantly in the IL-2 treated mice in any of the three tested conditions (A- no alemtuzumab, B- 8 weeks

post- alemtuzumab, and C- 2 weeks post alemtuzumab). The exception is a decrease in NK cells at week 2 (in both the control and IL-2 groups) and a decrease in CD4 T-cells at week 8 (control group only). These shifts are most likely a consequence of immune reconstitution post-alemtuzumab. Shifts in population frequencies reflect a combination of proliferation, survival/apoptosis and cell migration. The week 2 period is an inflexion point (at this time T-cell lymphopaenia starts to resolve, and Treg enrichment disappears by week 4- see Main Figure 4). Immune reconstitution related shifts slow down considerably by week 8 (when T-cell lymphopaenia is no longer statistically significant), but they likely do not stop entirely. In humans, immune reconstitution likewise proceeds with an initial fast phase, followed by a slower prolonged second phase (see ref. 4 for more detail).

Supplementary Figure 11: Comparison of Treg expansion in patients with type 1 diabetes and in MS post-alemtuzumab following the same IL-2 dosing regimen

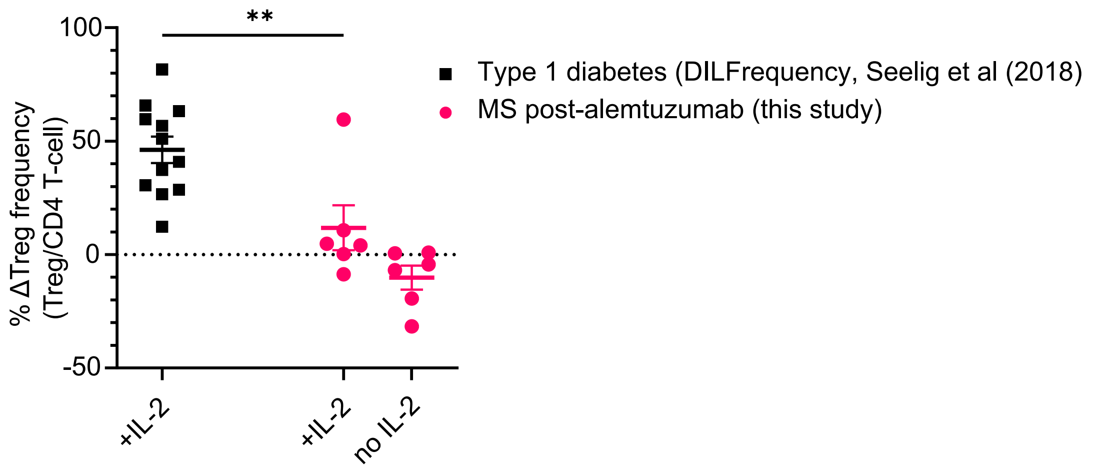

DILfrequency (Seelig et al JCI Insight 2018) was an adaptive dose-finding pharmacodynamic study of low-dose IL-2 in type 1 diabetes. The 12 individuals shown here all received 6 doses of IL-2 (Proleukin) at $0.32*10^6$ IU/m^2 every three days. Treg frequency was measured before and at the end of 6 doses, using the same methodology as for our study (LITMUS). See reference 25.

The LITMUS MS post-alemtuzumab patients received a nearly identical dose ($0.3*10^6$ IU/m^2 twice/week) and the same number (six) of Proleukin injections. For MS, Treg frequency was measured twice before IL-2, and again after completing six IL-2 injections. This is indicated as "no IL-2" and "+IL-2" respectively in this figure. For both studies the % Change in Treg frequency is calculated as:

% Δ= 100*(%Treg[end] - %Treg[baseline]/ %Treg[baseline]).

Two-tailed Mann Whitney test between the MS "on IL-2" (N=6) and diabetes (N=12) cohorts, p= 0.0069.

Supplementary Figure 12: Treg subpopulations during IL-2 dosing in the mechanistic study in MS patients post-alemtuzumab

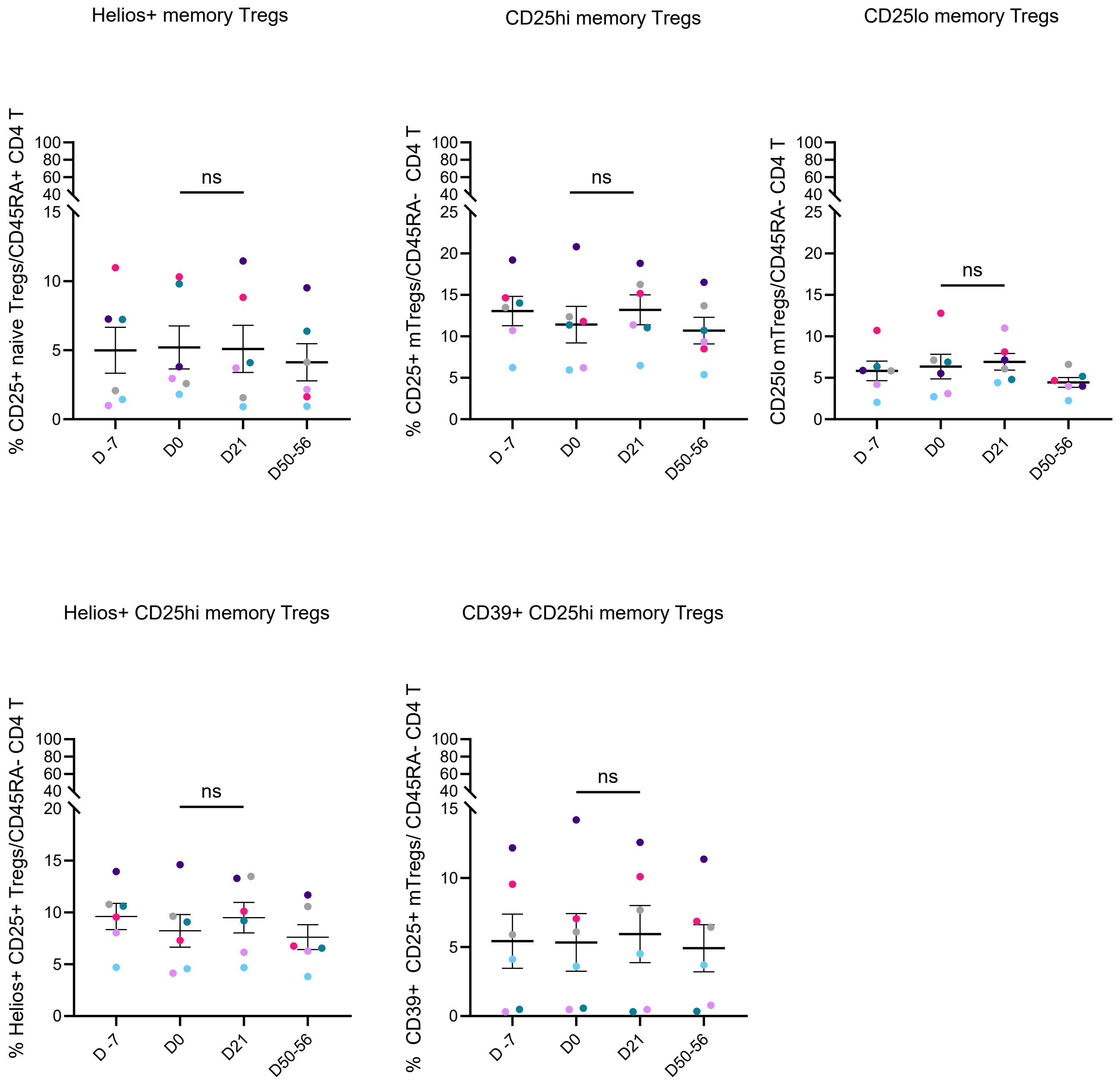

Additional Treg parameters measured pre/post IL-2 dosing in the MS mechanistic study. No changes were observed in any of these Treg sub-populations, some of which have previously been reported to expand with low-dose IL-2. Data points are coloured by individual donor (corresponding to main figure 7).
