# Supplementary material for: Low-dose IL-2 fails to expand Tregs after alemtuzumab: insight into impaired immune tolerance in multiple sclerosis
Source: Brain. 2026 May 13;149(11):4035–51. doi: 10.1093/brain/awag171 (PMC13730587; doi:10.1093/brain/awag171)
Supplement: Supplementary file 1 [file awag171_supplementary_data.zip › brain-2025-03236-File011.pdf]

Supplementary Table I Donor samples used for *in vitro* assays			
**Assay**	**Cohort type**	**Donor type**	**Numbers/groups**
Quantitative cytometry	Cross-sectional	RRMS pre & post-alemtuzumab	N= 10, Pre-alemtuzumab<br>N= 9, Month 6<br>N= 9, Month 9<br>N= 10, Month 12
pSTAT5	Longitudinal	RRMS pre- & post-alemtuzumab	N= 6 at these time points:<br>Pre-alemtuzumab Month 6-9 (combined)*<br>Month 12<br>Month 18-21 (combined)*
Serum IL-2†	Longitudinal	RRMS pre- & post-alemtuzumab	N= 9 at these time points:<br>Pre-alemtuzumab<br>Month 6<br>Month 12<br>Month 18<br>Month 24<br>Month 36
Spectral Treg phenotyping†	Longitudinal	RRMS pre- & post-alemtuzumab	N= 9 at these time points:<br>Pre-alemtuzumab Month 6-9 (combined)*<br>Month 12<br>Month 18-21 (combined)*<br>Month 36
TSDR demethylation	Cross-sectional	Healthy controls N=5<br>RRMS pre- & post-alemtuzumab	N= 5 HC<br>N= 6, pre-alemtuzumab N= 5, month 1-3<br>N= 3, month 6<br>N=6, month 12
Nanostring	Cross-sectional	Untreated MS<br>RRMS 6 months post-alemtuzumab	N= 4 Untreated MS<br>N= 4, 6 months post-alemtuzumab

*Where indicated, PBMC samples from two adjacent time points were combined to achieve the required cell number. † These are the same 9 individuals whose serum IL-2 level was measured.

Throughout the manuscript, time points after alemtuzumab are given in relation to the first treatment, e.g. “18 months” refers to 6 months after the second treatment (treatments are 12 months apart). In Figure 3, “month 6” and “month 18” data points have been treated as equivalent and designated “6 months after a treatment” for the purpose of generating dose-response curves.

SUPPLEMENTARY TABLE 2:

ANTIBODIES FOR IMMUNOPHENOTYPING AND CELL SORTING

**CD25/CD122 immunophenotyping**					
**Marker**	**Fluorochrome**	**Clone**	**Isotype**	**Catalogue N**	**Supplier**
CD3	BUV737	UCHT1	IgG1, κ	564307	BD
CD4	BUV395	SK3	IgG1, κ	563550	BD
CD8	BV650	RPAT8	IgG1,k	301042	Biolegend
CD56	BV605	HCD56	IgG1,k	318334	Biolegend
CD16	AF700	3G8	IgG1,k	302026	Biolegend
CD19	PECy7	HIB19	IgG1,k	302215	Biolegend
CD127	FITC	eBioRDR5	IgG1, k	11127842	Invitrogen
CD45RA	BV786	HI100	IgG2b, κ	563870	BD
CD27	V500	MT271	IgG1, κ	561222	BD
CD25	PE	ma251	IgG1,k	356104	Biolegend
CD25	PE	2A3	IgG1, κ	341011	BD
CD122	BV421	Mikb3	IgG1, κ	562887	BD
CD122	BV421	TU27	IgG1, κ	339010	Biolegend
FOXP3	AF647	259D	IgG1	560045	BD
HELIOS	PerCPCy5.5	22F6	Armenian hamster IgG	137230	Biolegend

**Treg spectral cytometry immunophenotyping**					
**Marker**	**Fluorochrome**	**Clone**	**Isotype**	**Catalogue N**	**Supplier**
CD3	BUV496	SK7	IgG1, κ	741206	BD
CD4	NovaFluor610/70S	SK3	IgG1,k	H001T03B06	ThermoFisher
CD8	PerCP	SK1	IgG1, k	344708	Biolegend
CD56	BUV563	NCAM16.2	IgG2b, κ	612929	BD
CD19	BB700	SJ25C1	IgG1, κ	566396	BD
CD14	cFluorB548	63D3	IgG1, k	R720115	Cytek
CD45RA	BUV395	HI100	IgG1, κ	740296	BD
CD27	APC	MT271	IgG1, k	356410	Biolegend
CD127	APCR700	HIL7RM21	IgG1, κ	565185	BD
CD25	BV421	MA251	IgG1, k	356114	Biolegend
CD25	BV421	2A3	IgG1, κ	564033	BD
CCR4	BV605	L291H4	IgG1, κ	359418	Biolegend
CCR6	BV650	G034E3	IgG2b, k	353426	Biolegend
CXCR3	PECy7	G025H7	IgG1, k	353720	Biolegend
CXCR5	SparkNIR685	J252D4	IgG1, k	356947	Biolegend
CD39	BUV661	TU66	IgG2b, κ	749967	BD
CD73	BUV805	AD2	IgG1, κ	748584	BD
CD226	BV510	11A8	IgG1, κ	338330	Biolegend
HLADR	BV570	L243	IgG2a, k	307638	BD
PD-1	BV711	EH12.1	IgG1, κ	564017	BD
GITR	BB515	V27-580	IgG2b, κ	567781	BD
TIGIT	PerCPefluor710	MBSA43	IgG1, κ	46950042	eBioscience
FOXP3	PE	PCH101	IgG2a, κ	12477642	eBioscience
FOXP3	PE	259D	IgG1, κ	320208	Biolegend
HELIOS	PE-Dazzle594	22F6	Armenian Hamster IgG	137232	BD
CTLA-4	PECy5	BNI3	IgG2a, κ	555854	BD
Ki67	PECy5.5	20Raj1	IgG1, κ	355699-41	eBioscience

PhosFlow pSTAT5 detection					
**Marker**	**Fluorochrome**	**Clone**	**Isotype**	**Catalogue N**	**Supplier**
CD3	BUV395	UCHT1	IgG1, κ	563546	BD
CD4	FITC	RPAT4	IgG1, κ	555346	BD
CD8	BUV737	SK1	IgG1, κ	612754	BD
CD56	PECy7	HCD56	IgG2a,k	318318	BD
CD45RA	BV786	HI100	IgG2b,k	304140	Biolegend
CD25	BB700	ma251	IgG1, κ	566447	BD
FOXP3	PE	236A/E7	IgG1, κ	12477742	Invitrogen
pSTAT5	AF647	pY694	IgG1, κ	612599	BD

Mouse immunophenotyping					
**Marker**	**Fluorochrome**	**Clone**	**Isotype**	**Catalogue N**	**Supplier**
TCRβ	PerCPCy5.5	H57597	Armenian Hamster IgG	109228	Biolegend
CD4	FITC	GK1.5	IgG2b, k	11004181	eBioscience
CD4	APC	GK1.5	Rat IgG2b,k	100412	Biolegend
CD8	BV605	53-6.7	Rat IgG2a, κ	100744	Biolegend
NKp46	PECy7	29A1.4	Rat IgG2a,k	137618	Biolegend
CD25	BV421	PC61	Rat IgG1, λ	102034	Biolegend
CD25	biotin	PC61.5	Rat IgG1 λ	13025185	eBioscience
CD11b	Pacific Blue	M1/70	Rat IgG2b, κ	101224	Biolegend
CD19	APCFire750	6D5	Rat IgG2a,k	115558	Biolegend
CD49b	PECy7	DX5	Rat IgM, κ	108922	Biolegend
CD11c	efluor450	N418	Armenian Hamster IgG	117322	Biolegend
Gr1 (Ly6C/ Ly6G)	Pacific Blue	RB6-8C5	Rat IgG2b, κ	108430	Biolegend
F4-80	BV421	BM8	Rat IgG2a, κ	123131	Biolegend
CD45.2	Biotin	104	MouseSJL IgG2a, κ	553771	BD
Ki67	Alexa Fluor 488	11F6	Rat IgG2b,k	151204	Biolegend
FoxP3	PE	FJK-16a	12577382, IgG2a, k		eBioscience
Human CD52	FITC	YTH34.5	MCA1642F, Rat IgG2b		BioRAD
Streptavidin	PE	-		554061	BD
Streptavidin	FITC	-		405202	Biolegend

LITMUS study- Clinical grade cytometry				
**Marker**	**Fluorochrome**	**Clone**	**Format**	**Supplier**
CD3	FITC	SK7	Cocktail	BD
CD16	PE	B73.1	Cocktail	BD
CD56	PE	NCAM16.2	Cocktail	BD
CD127	PE-dazzle 594	A019D5	Drop-in	Biolegend
CD45	PerCP-Cy5.5	2D1	Cocktail	BD
CD4	PE-Cy7	SK3	Cocktail	BD
CD19	APC	SJ25C1	Cocktail	BD
CD8	APC-Cy7	SK1	Cocktail	BD
CD25	BV421	2A3	Drop-in	BD
CD25	BV421	M-A251	Drop-in	Biolegend

LITMUS study- intracellular cytometry				
**Marker**	**Fluorochrome**	**Clone**	**Format**	**Supplier**
CD45	Krome Orange	J33	Duraclone	Beckman Coulter
CD4	PE-Cy7	SFCI12T4D11(T4)	Duraclone	Beckman Coulter
CD3	APC-A750	UCHT1	Duraclone	Beckman Coulter
CD56	PE-CF594	B159	Drop-in	BD
CD16	AF700	3G8	Drop-in	BD

CD25	PE	M-A251	Drop-in	BD
CD25	PE	B1.49.9	Duraclone	Beckman Coulter
CD39	PECy5.5	BA54	Duraclone	Beckman Coulter
HELIOS	Pacific Blue	22F6	Duraclone	Beckman Coulter
CD45RA	FITC	2H4LDH11LDB9(2H4)	Duraclone	Beckman Coulter
FOXP3	Alexa Fluor 647	259D	Duraclone	Beckman Coulter

Additional reagents, buffers and media

Reagent	Purpose	Catalogue N	Supplier
Ficoll Paque Plus	PBMC isolation	17-1440-03	Fisher Scientific
XVIVO-15	Thawing stored PBMCs	LONZ02-060F	VWR
Cell Freezing Media	Cell storage	C6164	Merck
FcR block	Cytometry- blocking	130-059-901	Miltenyi
TrueStain Monocyte Block	Cytometry- blocking	426102	Biolegend
Zombie Fixable viability dye	Viability discrimination	423106	Biolegend
Live Dead Blue	Viability (spectral)	L34962	ThermoFisher
FOXP3 TF Buffer Set	Intracellular staining	00-5523-00	Invitrogen
TF Buffer set	PhosFlow- fixation	562574	BD Biosciences
PhosFlow Perm Buffer III	PhosFlow- permeabilisation	558050	BD Biosciences
Qiagen Epitect Fast Bisulfite	Bisulfite conversion	59864	Qiagen
Zymo Quick RNA MicroPrep	RNA isolation	R1050	Cambridge Bioscience

Participant	Age	Sex	BSA (m^2)	RRMS duration (years)	Last recorded EDSS	Interval since last alemtuzumab (months)	Total number of alemtuzumab treatments	Autoimmunity
1	46	F	2.44	10	3.5	9-12	3	Yes, 2019
2	35	F	2.19	24	3.5	9-12	5	Yes, 2007 (resolved)
3	34	F	1.69	5	2.0	9-12	3	Yes, 2020
4	27	M	1.93	1	2.5	6-9	1	No
5	38	M	1.86	11	1.0	12	3	Yes, 2017
6	53	M	2.50	8	0.0	12	3	Yes, 2018

Supplementary Table 3. Patient characteristics in the experimental medicine study.

Supplementary Table 4 Clinical MS activity and autoimmunity after study completion

Participant	Time since last MS relapse at enrolment (weeks)	New MS activity after study completion	Time to new MS activity (weeks)	Autoimmunity at enrolment	New autoimmunity after study completion	Time to new autoimmunity (weeks)
1	106	No	-	Yes	No	-
2	76	Yes	97	Yes	No	-
3	91	No	-	Yes	Yes (haemorrhagica bullosa)	41
4	114	No	-	No	Yes (hypothyroidism)	89
5	143	Yes	30	Yes	No (reactivation of Graves disease)	61
6	148	Yes	147	Yes	No	-
Average (+/- SEM)	113+/13		91 +/- 41.5			64 +/-17
